# *In silico* analyses of conservational, functional and phylogenetic distribution of the LuxI and LuxR homologs in Gram-positive bacteria

**DOI:** 10.1038/s41598-017-07241-5

**Published:** 2017-08-01

**Authors:** Akanksha Rajput, Manoj Kumar

**Affiliations:** Bioinformatics Centre, Institute of Microbial Technology, Council of Scientific and Industrial Research, Sector 39A, Chandigarh, 160036 India

## Abstract

LuxI and LuxR are key factors that drive quorum sensing (QS) in bacteria through secretion and perception of the signaling molecules e.g. N-Acyl homoserine lactones (AHLs). The role of these proteins is well established in Gram-negative bacteria for intercellular communication but remain under-explored in Gram-positive bacteria where QS peptides are majorly responsible for cell-to-cell communication. Therefore, in the present study, we explored conservation, potential function, topological arrangements and evolutionarily aspects of these proteins in Gram-positive bacteria. Putative LuxI/LuxR containing proteins were retrieved using the domain-based strategy from InterPro *v*62.0 meta-database. Conservational analyses *via* multiple sequence alignment and domain showed that these are well conserved in Gram-positive bacteria and possess relatedness with Gram-negative bacteria. Further, Gene ontology and ligand-based functional annotation explain their active involvement in signal transduction mechanism *via* QS signaling molecules. Moreover, Phylogenetic analyses (LuxI, LuxR, LuxI + LuxR and 16s rRNA) revealed horizontal gene transfer events with significant statistical support among Gram-positive and Gram-negative bacteria. This *in-silico* study offers a detailed overview of potential LuxI/LuxR distribution in Gram-positive bacteria (mainly Firmicutes and Actinobacteria) and their functional role in QS. It would further help in understanding the extent of interspecies communications between Gram-positive and Gram-negative bacteria through QS signaling molecules.

## Introduction

LuxI and LuxR are the major component of quorum sensing (QS) based *lux* operon^1^. The basic mechanism of QS involves the secretion (LuxI) and perception (LuxR) of signaling molecules among microbes^2, 3^. Amongst them majorly exploited quorum sensing signaling molecules (QSSMs) for transmission are N-Acyl homoserine lactones (AHLs)^4, 5^, which are widely distributed in Gram-negative but with few reports of their presence in archaea^6^ and Gram-positive bacteria^7^.

Generally, bidirectionally transcribed *lux* operon (~218 bp distant) of *V*. *fischeri* comprised of 8 *lux* genes *luxA-E*, *luxG*, *luxI*, and *luxR* ^3^. LuxI protein is an acyl synthase of ~190 amino acid, secretes AHLs by catalyzing the reaction between S-adenosylmethionine (SAM) and acyl carrier protein (ACP)^8^. LuxR is an AHL recipient protein (252 amino acids) with N and C-terminal domains. Autoinducer binding domain (ABD) constitutes N-terminal region whereas DNA binding, helix-turn-helix (HTH) domain forms C-terminal region of LuxR regulator^3^. ABD recognizes and binds to respective AHL molecule. This complex promotes unmasking of C-terminus (DNA binding domain), stimulate its binding to DNA and activates transcription of various QS-controlled genes^2^.

Distribution of LuxI/LuxR proteins in the Gram-negative bacteria is well-characterized e.g. in *Vibrio fischeri* and *Vibrio harveyi* ^9^, *Pseudomonas aeruginosa* ^10^, *Erwinia* spp^1^. and many more as compiled in SigMol repository by our group^5^. There are several reports to explore the distribution and evolutionary history of LuxI/LuxR in Gram-negative bacteria^11, 12^ and their specific clades e.g. *Aeromonas* ^13^, *Roseobacter* ^14^, *Halomonadaceae* ^15^ and *Vibrionaceae* ^16^. However, few studies were performed for orphan LuxR (or LuxR solos) i.e. regulators that contain ABD (N-terminal) and DNA binding-HTH C-terminal domain but lack their cognate LuxI^17, 18^. Furthermore, recently we have performed computational exploration of LuxR solos in Archaea^19^.

Gram-positive bacteria primarily receive signals through QS peptides, that employed two-component system to complete the cascade^20^ rather than LuxI/LuxR homologs. Wynendaele *et al*. reported QS peptides from 51 Gram-positive bacteria in Quorumpeps database^21^. Subsequently, we have analyzed and predicted these QS peptides through various machine learning techniques in QSPpred web server^22^. In 2013, Biswa and Doble have shown the production of oxo-octanoyl homoserine lactone in a novel strain of *Exiguobacterium* sp., a marine Gram-positive bacterium^7^. This strain possesses a LuxR homolog designated as ExgR and also has LuxI homolog downstream to ExgR. Further, Bose *et al*. reported the production of N-(3-oxodecanoyl)-L-homoserine lactone and N-(3-oxododecanoyl)-L-homoserine lactone in *Salinispora* sp. (sponge associate marine Actinobacteria)^23^. Moreover, few genome annotation studies showed the presence of LuxI/LuxR in Gram-positive bacteria namely *Staphylococcus* spp., *Bacillus* spp., *Mycobacterium* spp., etc^24–26^. Actinobacteria phylum was phylogenomically explored by Santos and coworkers for LuxR regulators^27^ and later reviewed by Polkade *et al*. for the presence of possible QS^28^.

Gram-positive bacteria have two major phyla namely Actinobacteria (high G + C content) and Firmicutes (low G + C content). Amongst them, Firmicutes and other minor phylum were not explored for AHL-based intercellular communication. However, the presence of LuxI/LuxR in Gram-positive bacteria, strengthen the concept of interspecies communication between its species and that of Gram-negative bacteria. Therefore, in the present study we are analyzing complete Gram-positive bacteria group for the presence of putative LuxI/LuxR employing multidimensional perspectives like conservation, domain, motif, compositional, Gene ontology (GO), ligand-binding, clustering and taxonomic distribution. Notably, we also accomplished the evolutionary analyses for the occurrence of potential LuxI/LuxR in Gram-positive bacteria.

## Results

### Data analysis

**Figure 1:**
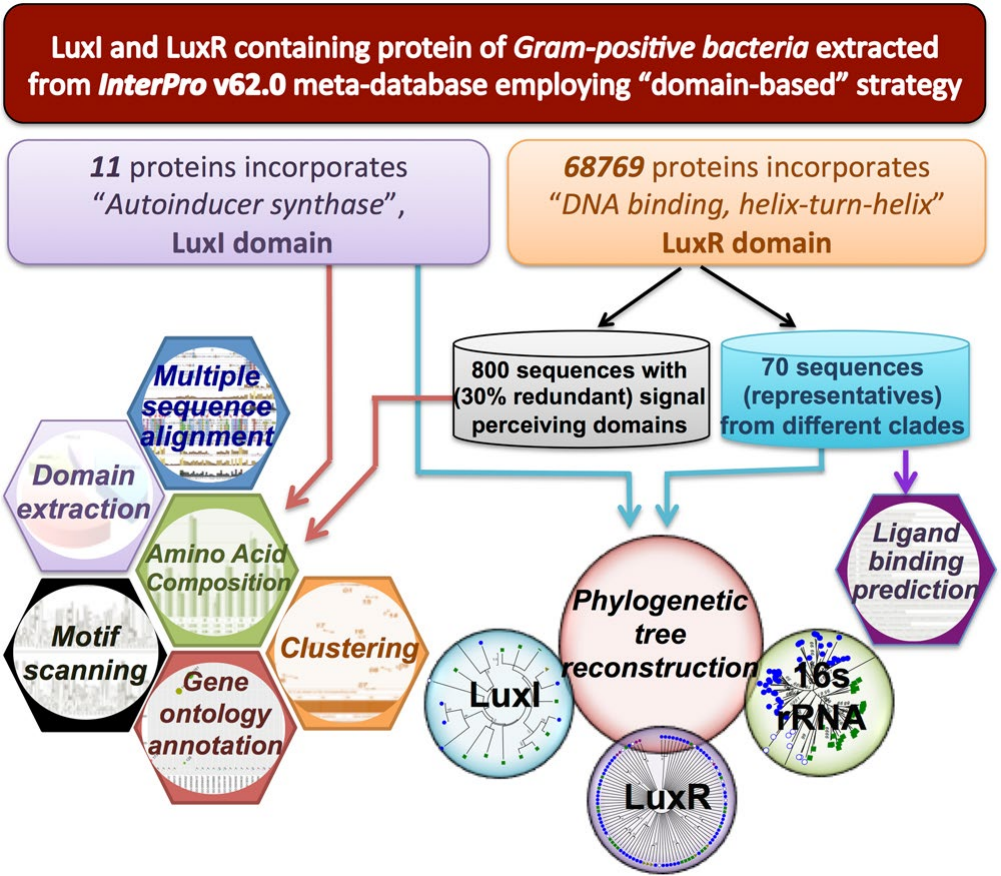
A flowchart depicting the amount of LuxI and LuxR containing proteins used in various analyses in the study.

LuxI and LuxR containing proteins of Gram-positive bacteria used in various analyses are schematically shown in Fig. 1. Further, we analyzed the length distribution (minimum, maximum and average) for both the categories of proteins. LuxI (11) containing proteins of Gram-positive bacteria had mean length of 219 amino acids. Proteins of *Asanoa ferruginea* (Micromonosporaceae family) and *Streptomyces purpurogeneiscleroticus* (Streptomycetaceae family) exhibit minimum length of 191 residues and *Ktedonobacter racemifer* (Ktedonobacteraceae family) incorporates a maximum length of 292 residues. Whereas, the 800 LuxR containing proteins showed an average length of 248 residues with minimum and maximum length of 200 and 300 correspondingly.

### Amino Acid Composition

We checked the amino acid composition (AAC) of putative LuxI and LuxR containing protein of Gram-positive bacteria and compared it with Gram-negative bacteria. For LuxI incorporating sequences amino acids like N and M were depleted in Gram-positive bacteria with the fold change of 0.66 and 0.80 respectively (*p value* < 0.05) (Supplementary Figure S1(a)). Whereas for LuxR, preferred and depleted residues are A and W with a fold change of 1.37 and 0.71 correspondingly (*p value* < 0.05) (Supplementary Figure S1(b)).

### Motif scanning

Motifs from LuxI and LuxR containing sequences of Gram-positive bacteria were scanned employing GLAM2 software and further searched in Gram-negative bacteria using GLAM2SCAN. Top 10 motifs from LuxI containing sequences were extracted that varied in width, sequence coverage and total alignment score (TAS) from 50–24, 11–08 and 387.12–70.16 respectively (Supplementary Table S1). Amongst 10 motifs, *Motif 1* is 43 amino acids in length, present in 10 sequences out of 11 and possesses TAS of 387.12. Moreover, we found top 25 hits of LuxI motifs of Gram-positive bacteria in Gram-negative bacteria that belonged to species of Methylobacteriaceae and Burkholderiaceae family.

Motif from LuxR containing sequences of Gram-positive bacteria was extracted, with *Motif 1* of width 47, covers 799 sequences out of 800 with TAS of 50338.8. Remaining motifs ranges in width, coverage, and TAS ranges from 35–48, 798–799 and 50019.4–38610.1 (Supplementary Table S2). However, scanning of LuxR containing motif (extracted from Gram-positive bacteria) in Gram-negative bacteria resulted in top 25 hits from species of Xanthomonadaceae, Pasteurellaceae, Rhodanobacteraceae, Anaeromyxobacteracea families.

### Domain analyses

**Figure 2:**
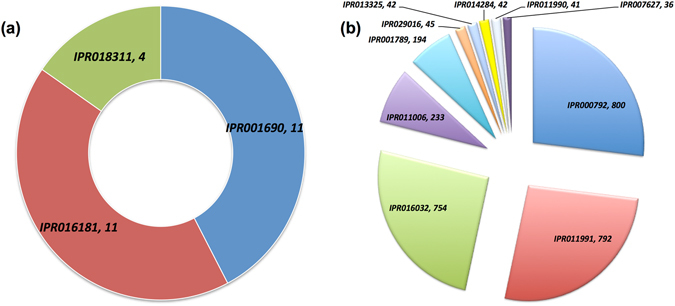
Statistical distribution of the domains that are maximum preferred (**a**) unique domains extracted in LuxI containing protein from InterPro, (**b**) unique domains extracted in LuxR containing protein from InterPro, *[IPR001690*, *Autoinducer synthase; IPR016181*, *Acyl-CoA N-acyltransferase; IPR018311*, *Autoinducer synthesis*, *conserved site; IPR000792*, *Transcription regulator LuxR*, *C-terminal; IPR011991*, *Winged helix-turn-helix DNA-binding domain; IPR016032*, *Signal transduction response regulator*, *C-terminal effector; IPR011006*, *CheY-like superfamily;IPR001789*, *Signal transduction response regulator*, *receiver domain; IPR029016*, *GAF domain-like; IPR013325*, *RNA polymerase sigma factor*, *region 2; IPR014284*, *RNA polymerase sigma-70 like domain; IPR011990*, *Tetratricopeptide-like helical domain; IPR007627*, *RNA polymerase sigma-70 region 2;]*.

Scanning of putative LuxI and LuxR incorporating proteins was done to extract all the possible domains. LuxI protein showed three hits amongst all the available domains in InterPro meta-database namely *Autoinducer synthase* (IPR001690), *Acyl-CoA N-acyltransferase* (IPR016181), and *Autoinducer synthesis*, *conserved site* (IPR018311) in 11, 11, and 04 sequences respectively (Fig. 2a). However, the combination of domains per protein was present in 07 and 04 sequences as *IPR016181* + *IPR001690* and *IPR016181* + *IPR018311* + *IPR001690* correspondingly. Moreover, NCBI-CDD reported only one domain with “*specific*” hit type i.e. Acetyltransf_5 in A0A1B1WGF3 protein of *Mycobacterium* sp. djl-10 (Mycobacteriaceae).

LuxR proteins displayed 78 unique hits from all the reported InterPro meta-database (Supplementary Table S3). Top hits of unique domains present in 800, 792, 754, 233, 194, 45, 42, 42 and 41 sequences belonged to *Transcription regulator LuxR*, *C-terminal* (IPR000792), *Winged helix-turn-helix DNA-binding domain* (IPR011991), *Signal transduction response regulator*, *C-terminal effector* (IPR016032), *CheY-like superfamily* (IPR011006), *Signal transduction response regulator*, *receiver domain* (IPR001789), *GAF domain-like* (IPR029016), *RNA polymerase sigma factor*, *region 2* (IPR013325), *RNA polymerase sigma-70 like domain* (IPR014284), *Tetratricopeptide-like helical domain* (IPR011990) respectively (Fig. 2b). However, the domain definition along with their homology with ABD or DNA binding domains for all the 78 unique domains is provided in Supplementary Table S4. Further, the combination of these 78 unique domains per protein resulted in 85 combinations (Supplementary Table S5). Maximum preferred domain combination is *IPR016032* + *IPR000792* + *IPR011991* in 339 sequences followed by *IPR011006* + *IPR016032* + *IPR001789* + *IPR000792* + *IPR011991*; *IPR016032* + *IPR011990* + *IPR000792 *+* IPR011991* and *IPR011006* + *IPR016032* + *IPR000792* + *IPR011991* in 179, 41 and 40 instances correspondingly. While searching the Gram-positive LuxR sequences using NCBI-CDD database we found unique 70 different domains as enlisted in Supplementary Table S6. Amongst them, *CitB*, *HTH_LUXR*, *LuxR_C_like* and *GerE* were maximally reported domains reported in 668, 646, 532, and 266 instances. From 70 unique domains, maximum 162-domain combinations were tabulated in Supplementary Table S7. Whereas, *CitB* + *HTH_LUXR* + *LuxR_C_like* + *GerE* followed by *CitB* + *HTH_LUXR* + *LuxR_C_like; CitB*; *HTH_LUXR* + *CitB* + *LuxR_C_like* present in 106, 92, 48 and 48 sequences correspondingly are amongst the maximally preferred domain combinations.

### Gene ontology

Putative LuxI/LuxR incorporating sequences were annotated for assignment of Gene Ontology (GO) domains namely molecular function, biological process and cellular component. LuxI sequences showed the presence of molecular function among three domains of GO. Out of eleven sequences, 9 were assigned with “*transferase activity*” (GO:0016740) and 1 with “*N-acetyltransferase activity*” (GO:0008080).

All the three GO domains were reported in 800 LuxR containing sequences of Gram-positive bacteria. LuxR proteins are reported in 09 different biological processes. Maximum sequences displayed “*regulation of transcription*, *DNA-templated*” (GO:0006355) followed by “*transcription*, *DNA-templated*” (GO:0006351), “*phosphorelay signal transduction system*” (GO:0000160), “*DNA-templated transcription*, *initiation*” (GO:0006352) in 712, 599, 184 and 54 instances. Pictorial representation of all 09 biological processes along with the number of LuxR protein sequences in which they are preferred are provided in Supplementary Figure S2(b). Further, exploring the proteins that assigned to be involved in the maximum biological process, we found A0A0U0JZZ2 (*Streptococcus pneumoniae* of Streptococcaceae family) exhibits five processes. However, LuxR containing proteins reported in 19 unique molecular functions with “*DNA binding*” (GO:0003677) as maximum favored among 773 sequences. Although, the “*transcription factor activity*, *sequence-specific DNA binding*” (GO:0003700), “*sigma factor activity*” (GO:0016987), “*phosphorelay sensor kinase activity*” (GO:0000155) testified in 55, 54, 05 proteins correspondingly (Supplementary Figure S2(b)). Maximum 05 molecular functions were assigned to A0A0U0N4G9 protein of *Streptococcus pneumoniae* (Streptococcaceae). Three unique cellular component i.e. “*intracellular*” (GO:0005622), “*integral component of membrane*” (GO:0016021), and “*ribosome*” (GO:0005840) exists in 189, 52 and 01 proteins respectively. Four LuxR containing proteins (A0A076JND1, C4FFF6, A0A1F8QL47, F6FQJ3) belonged to double cellular compartments (integral component of the membrane and intracellular) in the cell.

### Ligand-binding prediction

Identification of potential ligands that binds to LuxR regulators was accomplished using COACH software. We found that LuxR regulators of Gram-positive bacteria possess the ability to bind AHLs, peptides, Diffusible signal factors (DSFs), γ-butyrolactones, c-di-GMP, metals and many more (Supplementary Table S9). However, AHLs like N-(3-Oxo-octanal-1-yl)-homoserine lactone, N-Decanoyl-DL-homoserine lactone, N-3-Oxo-dodecanoyl-L-homoserine, N-Hexanoyl-L-homoserine lactone, etc. are predicted to bind with LuxR of Gram-positive bacteria. Moreover, DSFs like 3-Oxooctanoic acid and metals like Magnesium (+2), Manganese (+2), Copper (+2); Platinum (+2), etc. are identified to be recognized by response regulators of Gram-positive bacteria.

### Clustering

For grouping the related sequences we employed BLAST “*all-against-all*” pairwise similarity clustering approach. A gradient of *p-values* i.e. from relaxed (0.1) to more stringent one (1e-120) was employed to analyze the grouping pattern of proteins. For LuxI, two clusters were observed for 09 sequences out of 11 at *p-value* 1e-20 (Supplementary Figure S3(a)). While decreasing the *p-value* to 1e-120, only one cluster with two sequences of *Streptomyces purpurogeneiscleroticus* (Streptomycetaceae) (A0A0N0B975) and *Asanoa ferruginea* (Micromonosporaceae) (A0A0N0BAZ2) was reported.

Clustering of LuxR containing Gram-positive bacteria at a gradient of *p-value* ranging from 0.1 to 1e-60. For 800 Gram-positive bacteria at *p-value* 1e-45, 33 sequences congregated in 13 clusters with the species of Actinobacteria and Firmicutes phylum as depicted in Supplementary Figure S3(b). On decreasing the *p-value* to 1e-60 only two sequences remained grouped in single cluster.

### Multiple sequence alignment

**Figure 3:**
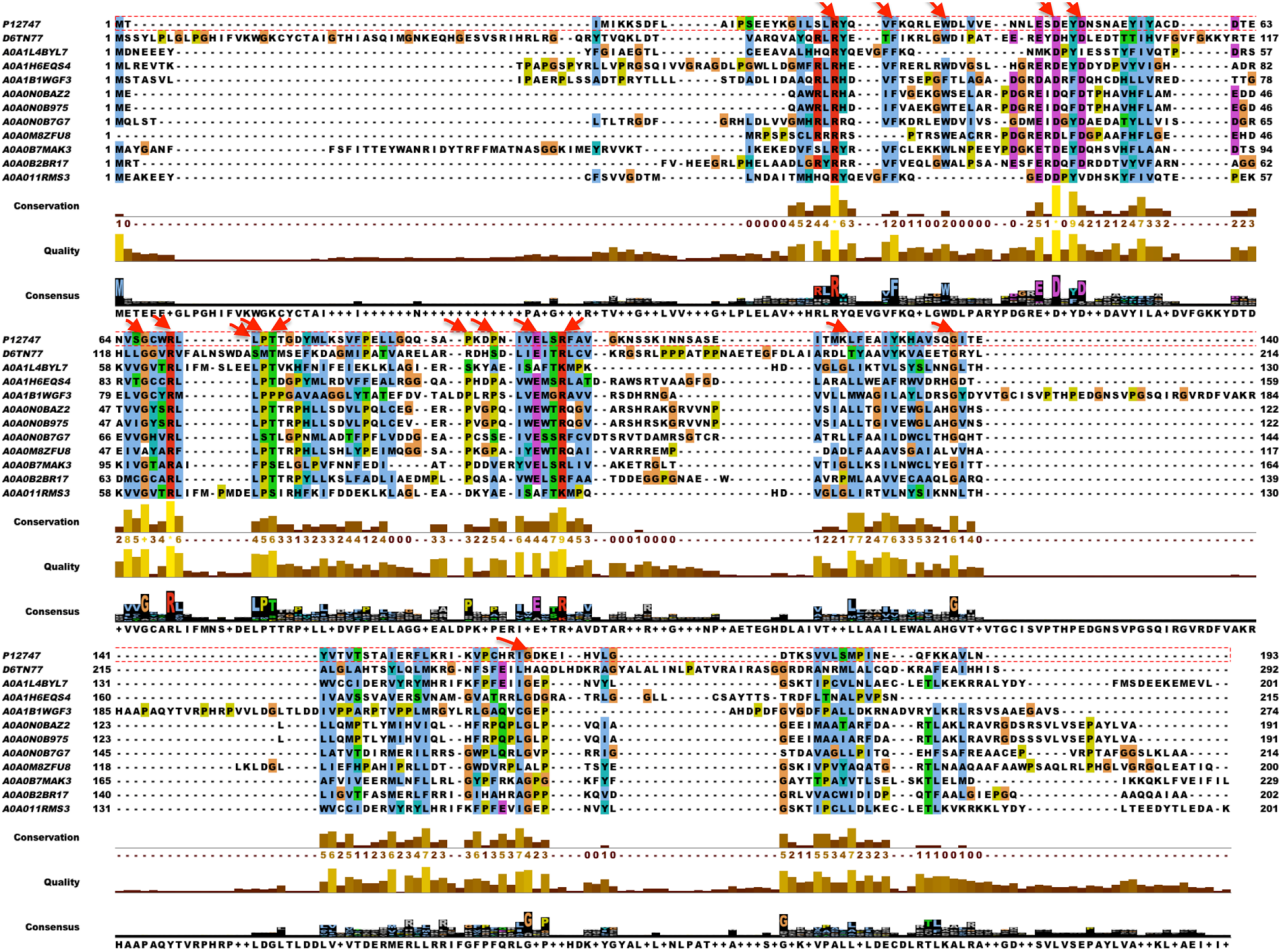
Multiple Sequence alignment of 11 LuxI containing sequences against *V*. *fischeri* LuxR sequence using MAFFT and visualized using Jalview software.

Evaluation for the invariant amino acid was performed for putative LuxI and LuxR against respective proteins of *V*. *fischeri* by multiple sequence alignment (MSA). LuxI incorporating sequences showed conservation among 33 amino acids with maximum in R25, F29, W35, E44, D46, D49, G67, R70, L72, P73, T74, P94, P97, E101, R104, L125, G137, G164 possessing identity of 100%, 92%, 75%, 83%, 100%, 83%, 92%, 100%, 83%, 83%, 75%, 75%, 50%, 83%, 83%, 75%, 83% and 83% respectively (Fig. 3). Information of all 33 conserved residues, position with gap insertion, percentage consensus and positions w.r.t. *V*. *fischeri* are shown in Supplementary Table S10. Whereas, LuxR containing sequences of Gram-positive bacteria displayed invariance in 17 amino acids and residues like L183, R186, E187, G197, I203, L207, T213, V214, K224, and R230 with consensus of 79.3%, 66%, 74,4%, 85.3%, 75.2%, 77.4%, 82.5%, 71.5%, 72.7% and 72.5% correspondingly showed maximum conservation (detailed in Supplementary Table S11).

### Topological arrangements of *luxI/luxR* genes

Topological arrangement of six canonical *luxI/luxR* is provided in Table 1. Adjacent *luxI* and *luxR* genes that transcribed in the same direction with $\overrightarrow{R}\overrightarrow{I}$ topological arrangement are found in *S*. *purpurogeneiscleroticus* (ADL19_05265/ ADL19_05260) and *A*. *ferruginea* (ADL14_01865/ ADL14_01860). While the oppositely transcribing direction of both the genes is present in *A*. *ferruginea* (ADL14_19790/ADL14_09775) with $\overrightarrow{R}\overleftarrow{I}$ topological arrangement. However, presence of some other genes i.e. X in between oppositely transcribing R and I e.g. $\overrightarrow{R}X\overleftarrow{I}\,$are found in *A*. *ferruginea* (ADL14_12710/ ADL14_22475) (X > 7) and *S*. *schinkii* (SSCH_1110008/SSCH_1100006) (X > 7), while in *M*. *flava* (LK11_10605/ LK11_10615) $\overleftarrow{I}X(2)\overrightarrow{R}\,$is reported.

**Table 1:** Topological arrangements of canonical *luxI/luxR* genes in Gram-positive bacteria.

Organisms	*luxI* locus tag(RefSeq)/Protein ID	*luxI* gene position	*luxR* locus tag(RefSeq)/ Protein ID	*luxR* gene position	Pattern	Gene Topology
*Asanoa ferruginea*	ADL14_12710/ A0A0M8ZFU8	3948..4550	ADL14_22475/ A0A0M8ZBK1	117..689	$\overrightarrow{R}X( > 7)\overleftarrow{I}$	
*Asanoa ferruginea*	ADL14_01865/ A0A0N0BAZ2	29123..29698	ADL14_01860/ A0A0N0U2C6	28172..28900	$\overrightarrow{R}\overrightarrow{I}$	
*Asanoa ferruginea*	ADL14_19790/ A0A0N0B7G7	49967..50611	ADL14_09775/ A0A0M8ZHM1	49208..49804	$\overrightarrow{R}\overleftarrow{I}$	
*Mumia flava*	LK11_10605/ A0A0B2BR17	94575..95183	LK11_10615/ A0A0B2BQK8	95914..96633	$\overleftarrow{I}X(2)\overrightarrow{R}$	
*Streptomyces purpurogeneiscleroticus*	ADL19_05265/ A0A0N0B975	38419..38994	ADL19_05260/ A0A0N0B8Y7	37479..38207	$\overrightarrow{R}\overrightarrow{I}$	
*Syntrophaceticus schinkii*	SSCH_1110008/ A0A0B7MAK3	4194..4883	SSCH_1100006/ A0A0B7MIQ5	3074..3310	$\overrightarrow{R}X( > 7)\overleftarrow{I}$	

## Phylogenetic analyses

### Phylogenetic exploration of LuxI and LuxR families

**Figure 4:**
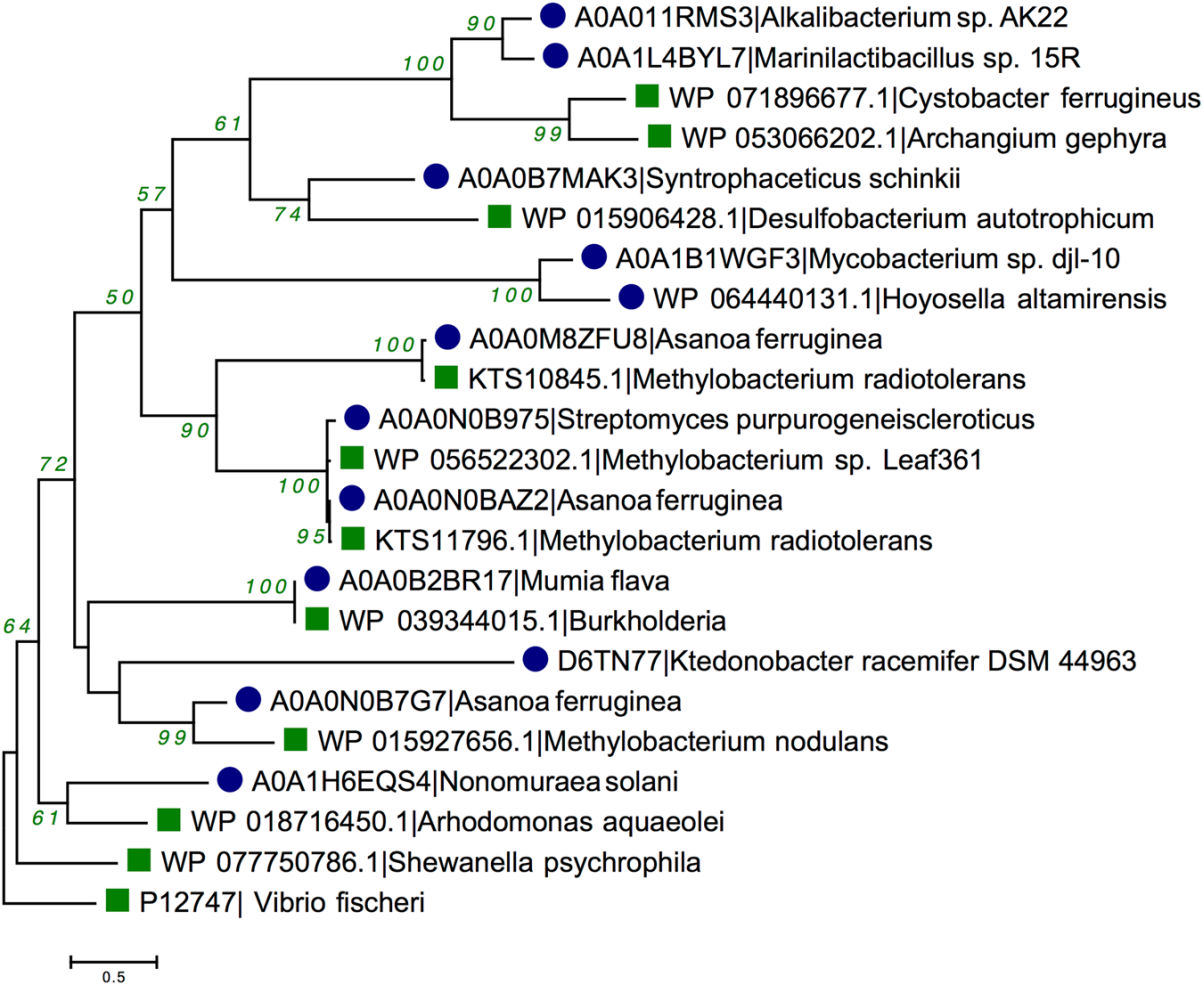
Phylogenetic tree reconstruction of LuxI containing protein employing Maximum Likelihood method on Gram-positive bacteria and their respective BLAST hits [*Gram-positive bacteria* (), and *Gram-negative bacteria* ()].

Reconstruction of phylogenetic trees was done to investigate the evolutionary trends in LuxI and LuxR proteins. The Maximum Likelihood (ML) method used for building the phylogenetic tree between LuxI and their respective BLAST hits to evaluate the gene transfer among Gram-positive bacteria. All the 11 LuxI sequences of Gram-positive bacteria located with their respective BLAST hits i.e. Gram-negative bacteria except *Mycobacterium* sp. djl-10 with high bootstrap support (Fig. 4). For example *Mumia flava* (Nocardioidaceae) with *Burkholderia* (Burkholderiaceae) (Bootstrap 100); *Streptomyces purpurogeneiscleroticus* (Streptomycetaceae) with *Methylobacterium* sp. Leaf361 (Methylobacteriaceae) (99); *Syntrophaceticus schinkii* (Thermoanaerobacterales Family III. IncertaeSedis) with *Desulfobacterium autotrophicum* (Desulfobacteraceae) (74), etc.

**Figure 5:**
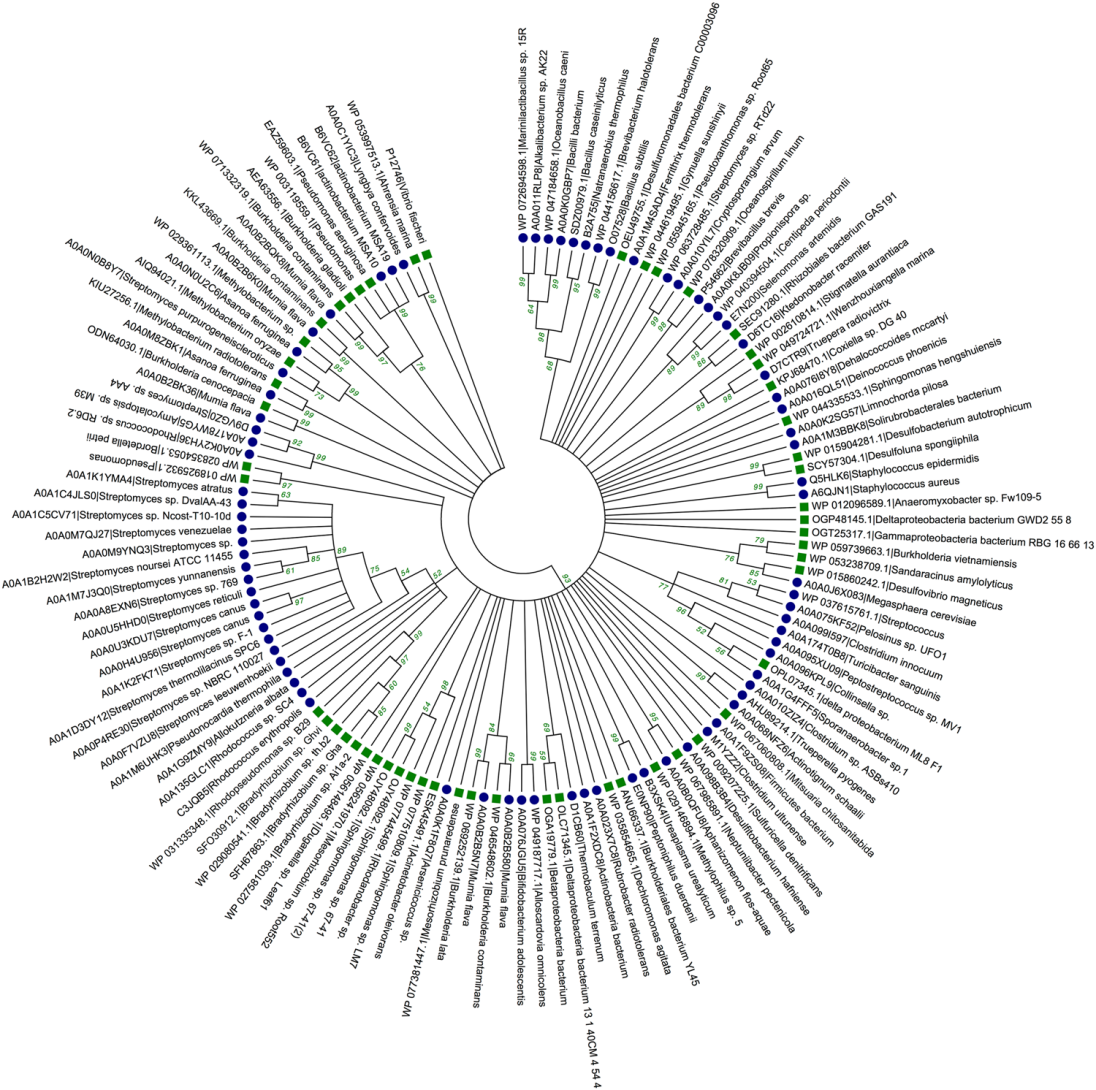
Phylogenetic tree reconstruction of LuxR containing protein employing Maximum Likelihood method on Gram-positive bacteria and their respective BLAST hits [*Gram-positive bacteria* (), and *Gram-negative bacteria* ()].

ML tree for representative LuxR sequences of Gram-positive along with their respective BLAST hits is provided in Fig. 5. It showed that maximum Gram-positive bacteria localized with Gram-negative bacteria with the exception of two groups possessing species of Streptomycetaceae family (*Streptomyces* spp.) and Bacillaceae and Lactobacillaceae family (*Bacillus* spp., *Alkalibacterium* sp., *Oceanobacillus caeni*, *etc*.). Few examples for colocalization of Gram-positive and Gram-negative bacteria with high bootstrap support includes *Brevibacillus brevis* (Paenibacillaceae) with *Oceanospirillum linum* (Oceanospirillaceae) (Bootstrap value 98); *Ktedonobacter racemifer* (Ktedonobacteraceae) and *Rhizobiales bacterium* GAS191 (unclassified Rhizobiales) (86); *Megasphaera cerevisiae* (Veillonellaceae) and *Desulfovibrio magneticus* (Desulfovibrionaceae) (85); *Aphanizomenon flos-aquae* (Aphanizomenonaceae) with *Neptuniibacter pectenicola* (Oceanospirillaceae) (95); *Mumia flava* (Nocardioidaceae) and *Burkholderia* spp. (Burkholderiaceae) (99), and many more as depicted in Fig. 5.

### Putative LuxI/LuxR cassette transfer pattern

**Figure 6:**
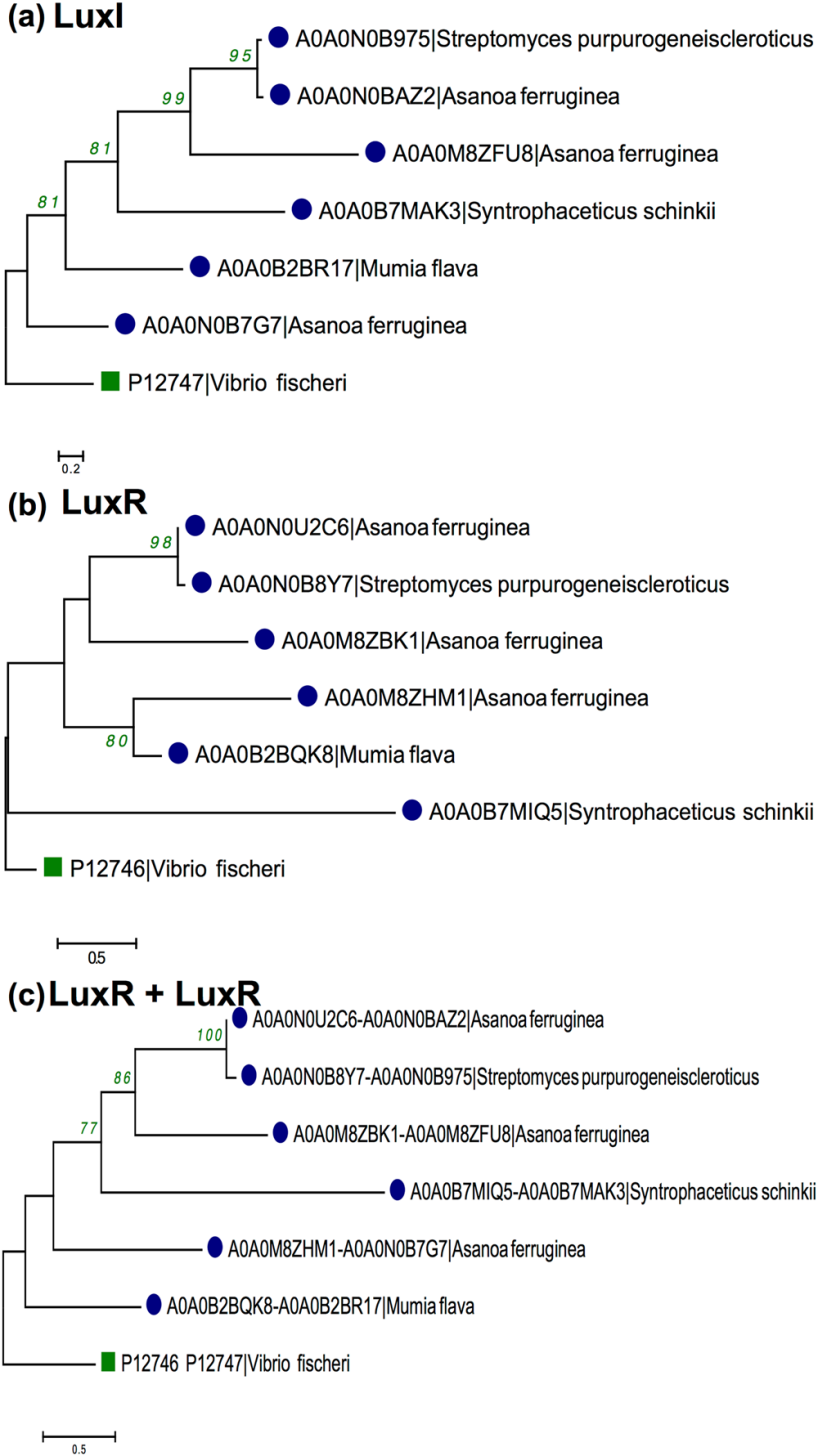
Phylogenetic tree reconstruction using Maximum Likelihood method for Gram-positive bacteria (**a**) LuxI containing sequences, (**b**) LuxR containing sequences, and (**c**) LuxR + LuxI sequences against *V*. *fischeri* LuxI and/or LuxR as outgroup. [*Gram-positive bacteria* (), and *Gram-negative bacteria* ()].

To observe the transfer pattern of 06 LuxI/LuxR cassettes in Gram-positive bacteria, phylogenetic trees were reconstructed using individual LuxI, LuxR and concatenated LuxI + LuxR. Three out of six QS proteins showed similar topology in all three trees namely *S*. *purpurogeneiscleroticus*, *A*. *ferruginea* (2 proteins). While the protein of *S*. *schinkii* is localized in the same clades between two trees (LuxI and LuxI + LuxR). Moreover, the positions of *M*. *flava* and one protein of *A*. *ferruginea* are not clear. The entire three phylogenetic reconstruction patterns are provided in Fig. 6.

AHL-based QS is a typical characteristic of Gram-negative bacteria but its presence in Gram-positive bacteria is questionable. Therefore, we reconstructed phylogenetic tree for 06 LuxI and their cognate LuxR in Gram-positive bacteria using the top-most hit of Gram-negative bacteria from BLAST similarity search. Phylogenetic tree for LuxI containing protein of Gram-positive and their respective Gram-negative bacteria are shown in Supplementary Figure S4(a). Every Gram-positive bacterium’s autoinducer synthase proteins positioned with Gram-negative bacteria with good bootstrap support. For example, *S*. *purpurogeneiscleroticus* and *Methylobacterium* sp. (99); *A*. *ferruginea* with *Methylobacterium* sp. Leaf361 (98); *M*. *flava* with *Burkholderia* (100) etc. Similar pattern was observed in LuxR containing protein, with good bootstrap support in between Gram-positive and Gram-negative bacteria as shown in Supplementary Figure S4(b) like *S*. *purpurogeneiscleroticus* and *Methylobacterium* sp. (68); *A*. *ferruginea* with *Methylobacterium* sp. Leaf361 (95); *M*. *flava* with *Burkholderia* (100), etc. When comparing the LuxI and LuxR containing protein with 16s rRNA gene tree, we found that Gram-positive and Gram-negative bacterial sequences clustered separately unlike LuxI and LuxR tree topology.

## Discussion

QS is an imperative phenomenon of intercellular communication among bacteria and driven by various QSSMs (AHL-based in Gram-negative bacteria, peptides for Gram-positive bacteria, etc.)^2, 29^. However, recently there are instances of interspecies, and interkingdom communication *via* signaling molecules^30, 31^. In this study, we tried to demonstrate the interspecies communication among Gram-positive and Gram-negative bacteria through various QSSMs especially AHLs. Bioinformatics survey was done according to conservational, functional, evolutionarily and taxonomic distribution of putative LuxI and LuxR proteins in complete Gram-positive bacteria (major phylum Actinobacteria and Firmicutes) and compared it with Gram-negative bacteria.

Gram-positive bacteria were scanned for the presence of putative LuxI/LuxR sequences through InterPro meta-database, which incorporate automatically annotated tools to produce signature to describe protein families employing HMM based criteria from associated databases. Gram-positive bacteria possess few instances of LuxI (11) whereas numerous LuxR solos (68769). Further, to check the presence of canonical LuxI/LuxR system we used the criteria mentioned in previous studies i.e. the distance between *luxI* and *luxR* (less than 3000 bp/3400 bp), length of ORF, LuxR incorporating ABD and DNA binding domain^32–34^, which resulted in six canonical LuxI/LuxR systems in Gram-positive bacteria. Surprisingly, for remaining five putative LuxI sequences of Gram-positive bacteria we could not identify cognate LuxR using the above distance criteria. Interestingly, LuxR solos sequences are available in these organisms beyond the above-mentioned distance. However, the presence of only six putative LuxI/LuxR pair indicates that Gram-positive bacteria possess less ability to secrete AHLs as compared to Gram-negative bacteria. Although, they can sense wide range on QSSMs including AHLs, DSFs, etc., due to the presence of numerous LuxR solos (LuxR that lacks cognate LuxI).

The topological arrangement of six canonical *luxI* and *luxR* genes among Gram-positive bacteria showed that some of them are similar to Gram-negative bacteria as adjacently transcribing locus e.g. $\overrightarrow{R}\overrightarrow{I}\,{\rm{and}}\,\overrightarrow{R}\overleftarrow{I}$found in proteobacteria (α, β, γ and θ)^32, 33^. Whereas, we also found some different topological arrangements in Gram-positive bacteria like and $\overleftarrow{I}X(2)\overrightarrow{R}$ and $\overrightarrow{R}X( > 7)\overleftarrow{I}$ that are not reported in Gram-negative bacteria till date.

The amino acid composition analysis between Gram-positive and Gram-negative bacteria showed that they both are considerably related to each other but with fewer differences (statistically significant) in amino acids. Further, we checked Gram-negative bacteria for the presence of LuxI/LuxR motifs (conserved patterns of amino acid) of Gram-positive bacteria. Top 10 LuxI and LuxR extracted motifs were rendered with PROSITE family profile and signature i.e. AUTOINDUCER_SYNTH_2 (PS51187), AUTOINDUCER_SYNTH_1 (PS00949) and HTH_LUXR_2 (PS50043), HTH_LUXR_1 (PS00622) respectively, which was previously reported in LuxI and LuxR sequences^35, 36^. Therefore, our motif analysis indicates that LuxI/LuxR of Gram-positive bacteria are similar to that of Gram-negative bacteria.

To analyze the independently existing portion of the protein with the specific function we performed domain extraction studies. Domains extracted using two strategies (InterPro and NCBI-CDD) revealed the preference of “*Autoinducer synthase*” for LuxI; “*response regulator binding*, *N-terminal*” and “*Transcription regulator LuxR*, *C-terminal*” for LuxR. The domains from LuxI containing protein of Gram-positive bacteria are related to autoinducer synthesis. For example, “*Autoinducer synthase*” (IPR001690, IPR018311) responsible for synthesizing AHLs by utilizing acyl-(acyl-carrier proteins) (acyl-ACP) and amino acids as substrates in the presence of acyl transferase (IPR016181, Acetyltransf_5)^37^. Likewise, among LuxR containing proteins i.e. both N-and C-terminal portion comprised of domains that complement them to complete the phenomenon of signal transduction maximally *via* two-component system (TCS) (characteristic of Gram-positive QS system)^27^. For example, amongst the preferred domains of C-terminal LuxR, mostly belonged to TCS (IPR000792, IPR016032, IPR011006, IPR001789) and exhibits the ability for binding to DNA *via* HTH loop (IPR000792, IPR011991) that further participates in transcription initiation and elongation (IPR013324, IPR014284, IPR013325)^38, 39^. Thus, domain analysis further supports that Gram-positive bacteria possess functional components for AHL based communication like that of Gram-negative bacteria.

Functional annotation of potential LuxI/LuxR incorporating proteins was accomplished using GO and ligand-based analysis. As LuxI containing proteins was assigned with “*transferase activity*”, which might helps Gram-positive bacteria to transfer acyl group during AHL synthesis^37, 40^. In case of LuxR, predominant biological processes involve in DNA dependent and gene-specific transcription. Molecular function assignment showed that proteins exhibit the ability for sequence-specific DNA binding along with sigma factor and phosphorelay sensor kinase activities that are the important component of bacterial signal transduction^41^. Further maximum activity of LuxR proteins reported to be localized in membrane and intracellular, proves their involvement in TCS. Therefore, GO annotation study indicates that Gram-positive bacteria display the ability to synthesize and responds towards AHLs as that of Gram-negative bacteria. Moreover, ligands prediction for LuxR sequences of Gram-positive bacteria showed that they possess the ability to bind to various QSSMs like bind AHLs, peptides, DSFs, γ-butyrolactones, c-di-GMP, etc. Despite the peptides are considered as the major signaling molecules in Gram-positive bacteria^22, 42^, the presence of QSSMs of Gram-negative bacteria like AHLs, DSFs were also reported. The interaction between Gram-positive and Gram-negative bacteria is supported by the presence of QSSMs like AHLs, DSFs, and γ-butyrolactones (structural homolog of AHLs)^5, 28^. Moreover, presence of ubiquitous signaling molecules i.e. c-di-GMP that might assist them to undergo phenotypic changes like virulence and biofilms^28^. Thus, the ligand binding prediction study further supports the existence of interspecies communication between Gram-positive and Gram-negative bacteria.

Clustering of the QS proteins of Gram-positive bacteria was executed to observe their assemblage pattern according to similarity. Grouping pattern at significant *p-values* using BLAST approach on LuxI, exhibited similarity among Gram-positive (Actinobacteria and Firmicutes) themselves. Likewise, for LuxR sequences, the same pattern of relatedness was observed at stringent *p-values*. Hence, clustering analysis indicates that LuxI and LuxR containing proteins distribution are in accordance to taxonomic lineages.

Consensus between LuxI and LuxR containing proteins of Gram-positive bacteria were extracted by aligning with Gram-negative bacteria (*V*. *fischeri*). MSA for the LuxI of Gram-negative bacteria against *V*. *fischeri* revealed that they possess high similarity with the critical residues of LuxI (with active site and substrate specificity (acyl-ACP) site) sequences. Moreover, eleven important residues were found conserved in them as reported to be key sites in LuxI family i.e. R25, F29, W35, D49, R70, R104, A133 and E150 with considerable sequence identity^43^. In the case of LuxR, most of the residues were found conserved in 800 representative Gram-positive bacteria species as that of LuxR family proteins. For example, L183, T184, R186, E187, L191, G197, I203, L207, T 213, V214, H217, K224 and R230 that are critical residues for DNA binding activity of LuxR regulator^36^. Thus, our alignment analysis proved that putative LuxI/LuxR in Gram-positive bacteria is similar to that of Gram-negative bacteria with critical residues intact.

The presence of two sequences from the different group in same branch with high bootstrap support along with the presence in same ecological niche and showed deviation from 16s rRNA gene tree confirms the presence of horizontal gene transfer (HGT)^19, 44^. The phylogenetic tree for LuxI sequences showed that 10 out of 11 LuxI sequences might have transferred horizontally that belonged to same ecological niche i.e. soil or plant-associate ecosystem between Gram-negative and Gram-positive bacteria. Subsequently, in LuxR regulators, most of the branching pattern depicts the HGT between both groups of species, which are also the inhabitant of same ecosystem e.g. soil and plant associated (*A*. *ferruginea*, *Methylobacterium* spp., *Mumia flava*, *Burkholderia* spp., etc), aquatic ecosystem (*Aphanizomenon flos-aquae* and *Neptuniibacter pectenicola*, *Oceanospirillum linum*), and many more. Hence, the evolutionary trend analysis signifies that majority of the LuxI and LuxR sequences of Gram-positive bacteria may have acquired through HGT from Gram-negative bacteria.

Transfer pattern of putative LuxI + LuxR cassette was checked employing phylogenetic analysis. We found that LuxI + LuxR cassette transferred simultaneously in *S*. *purpurogeneiscleroticus and A*. *ferruginea* (2 proteins). While the LuxI and LuxR of *S*. *schinkii* were transferred individually. Moreover, transfer pattern of QS proteins is unclear in *M*. *flava* and *A*. *ferruginea* (1 copy). Thus, the inheritance pattern analysis showed that in most of the Gram-positive bacteria complete LuxI + LuxR loci moved simultaneously followed by individual transfer of LuxI and LuxR. Further, we checked the source of potential canonical LuxI/LuxR in Gram-positive bacteria through phylogenetic analysis using respective Gram-negative bacteria in BLAST hit. On integrating top-most Gram-negative bacterial BLAST hit of LuxI and LuxR Gram-positive bacteria, we found that Gram-positive bacteria positioned with respective Gram-negative bacteria supported by good bootstrap values. Moreover, 05 out of 06 Gram-positive bacteria possess same hosts in both LuxI and LuxR and are the inhabitant of same ecological niche (Table 2). For example canonical LuxI/LuxR system from all three copies of *A*. *ferruginea* derived from *Methylobacterium* spp. (*Methylobacterium radiotolerans* and *Methylobacterium nodulans*) that are the resident of plant-associated ecosystem; *M*. *flava* found with *Burkholderia* spp. (Plant-associated); *S*. *purpurogeneiscleroticus* showed significant similarity with *Methylobacterium* sp. Leaf361 (Leaf surface). However, *S*. *schinkii* (aquatic) is the exception with BLAST hits of LuxI and LuxR from *Desulfobacterium autotrophicum* (aquatic) and *Sandaracinus amylolyticus* (soil) respectively from different habitats but same taxonomic group (Deltaproteobacteria). Hence, phylogenetic analysis confirmed the HGT of putative LuxI and LuxR sequences between Gram-positive and Gram-negative bacteria.

**Table 2:** Table displaying protein type, IDs, organism name, topological orientation, ecological niche and taxonomic details of Gram-positive bacteria (06 LuxI and their cognate LuxR) and their corresponding top-most BLAST hit Gram-negative bacteria.

Protein type	Proteins IDs	Bacteria	Strain	Taxonomy	Ecological niche
LuxI	A0A0M8ZFU8	*Asanoa ferruginea*	NRRL B-16430	Gram-positive (Actinobacteria)	Soil
BLAST hit	KTS10845.1	*Methylobacterium radiotolerans*	SB3	Gram-negative (Alphaproteobacteria)	Plant-associated
LuxI	A0A0N0BAZ2	*Asanoa ferruginea*	NRRL B-16430	Gram-positive (Actinobacteria)	Soil
BLAST hit	KTS11796.1	*Methylobacterium radiotolerans*	SB3	Gram-negative (Alphaproteobacteria)	Plant-associated
LuxI	A0A0N0B7G7	*Asanoa ferruginea*	NRRL B-16430	Gram-positive (Actinobacteria)	Soil
BLAST hit	WP_015927656.1	*Methylobacterium nodulans*	na	Gram-negative (Alphaproteobacteria)	Plants (rhizoplane)
LuxI	A0A0B2BR17	*Mumia flava*	MUSC 201	Gram-positive (Actinobacteria)	Plants (rhizosphere)
BLAST hit	WP_039344015.1	*Burkholderia*	na	Gram-negative (Betaproteobacteria)	Agriculture field soil
LuxI	A0A0N0B975	*Streptomyces purpurogeneiscleroticus*	NRRL B-2952	Gram-positive (Actinobacteria)	Soil
BLAST hit	WP_056522302.1	*Methylobacterium sp*.	Leaf361	Gram-negative (Alphaproteobacteria)	Leaf surface
LuxI	A0A0B7MAK3	*Syntrophaceticus schinkii*	Sp3	Gram-positive (Firmicutes)	Waste water (aquatic)
BLAST hit	WP_015906428.1	*Desulfobacterium autotrophicum*	na	Gram-negative (Deltaproteobacteria)	Marine (sediment) (aquatic)
LuxR	A0A0M8ZBK1	*Asanoa ferruginea*	NRRL B-16430	Gram-positive (Actinobacteria)	Soil
BLAST hit	KIU27256.1	*Methylobacterium radiotolerans*	78c	Gram-negative (Alphaproteobacteria)	Plant-associated
LuxR	A0A0N0U2C6	*Asanoa ferruginea*	NRRL B-16430	Gram-positive (Actinobacteria)	Soil
BLAST hit	WP_076727804.1	*Methylobacterium radiotolerans*	na	Gram-negative (Alphaproteobacteria)	Plant-associated
LuxR	A0A0M8ZHM1	*Asanoa ferruginea*	NRRL B-16430	Gram-positive (Actinobacteria)	Soil
BLAST hit	WP_043074725.1	*Methylobacterium radiotolerans*	na	Gram-negative (Alphaproteobacteria)	Plant-associated
LuxR	A0A0B2BQK8	*Mumia flava*	MUSC 201	Gram-positive (Actinobacteria)	Plants (rhizosphere)
BLAST hit	WP_039344021.1	*Burkholderia*	na	Gram-negative (Betaproteobacteria)	Agriculture field soil
LuxR	A0A0N0B8Y7	*Streptomyces purpurogeneiscleroticus*	NRRL B-2952	Gram-positive (Actinobacteria)	Soil
BLAST hit	WP_056522117.1	*Methylobacterium sp*.	Leaf361	Gram-negative (Alphaproteobacteria)	Leaf surface
LuxR	A0A0B7MIQ5	*Syntrophaceticus schinkii*	Sp3	Gram-positive (Firmicutes)	Waste water (aquatic)
BLAST hit	WP_083458420.1	*Sandaracinus amylolyticus*	na	Gram-negative (Deltaproteobacteria)	Soil

AHL-based social networking is the typical feature of Gram-negative bacteria, but its presence in Gram-positive bacteria needs to be explored. The analyses done in the study revealed that QS regulatory cassette of Gram-positive bacteria (mainly Firmicutes and Actinobacteria) is acquired from Gram-negative bacteria through HGT simultaneously or individually. The HGT assists bacteria to adapt in novel ecological niche^45–47^. Moreover, there are the evidence of the transfer of complete metabolic operon in bacteria e.g. *lac* operon^47^. Further, the coexistence of Gram-positive and Gram-negative bacteria in multispecies or polymicrobial biofilms at oral or dental plaque^48, 49^, respiratory tract^50^, catheters^51^, surface of marine algae^52^ and many more further strengthen our findings. Although the instances for the occurrence of LuxR is very high as compared to LuxI that explain the extent for responding to QSSMs are very high as compared to synthesis in Gram-positive bacteria. Furthermore, AHLs might emerge as an active potential tool for the interspecies communication between Gram-positive bacteria and Gram-negative bacterial species. Simultaneously, the presence of AHL-based QS circuit in Gram-positive bacteria might help them to survive in the same ecological niche where Gram-negative bacteria are present by undergoing interspecies communication with them in addition to intraspecies communication through QSPs. Therefore, an updated quorum quenching strategies might be useful against bacteria in biofilm mode.

## Methods

### Data retrieval

LuxI and LuxR containing sequences were extracted from InterPro *v*62.0^53^. InterPro is a meta-database that integrates information from various sub-databases (CATH-Gene3D, TIGRFAMs, PROSITE patterns and profiles, Pfam, PANTHER, etc.) and provides them in less redundant and easily searchable form.

Domain-based search was done to fetch out the “*Autoinducer synthase*” (IPR001690) and “*Transcription regulator LuxR*, *C-terminal*” (IPR000792) incorporating proteins from Gram-positive bacteria that is major phyla of Terrabacteria taxon namely Firmicutes, Actinobacteria, Chloroflexi, Tenericutes, Cyanobacteria/Melainabacteria, Deinococcus-Thermus, Armatimonadetes, and unclassified Terrabacteria. The reported LuxI and LuxR containing sequences were 11 and 68769 respectively. Since, 68769 LuxR containing sequences were difficult to handle, so we used filters to get a significant number of sequences. Firstly, we extracted sequences with DNA binding domain and autoinducer (or ligand) binding domain as mentioned by Hudaiberdiev *et al*.^34^, which resulted in 45365 entries. Secondly, we utilized CD-HIT^54^ suite to choose representative sequence (800 proteins) having not more than 30% sequence identity. All the analyses were performed with these sequences to explore various aspects of their presence in Gram-positive bacteria. The flowchart depicting the LuxI and LuxR proteins used in various analyses are provided in Fig. 1. Moreover, the protein IDs of the LuxI and LuxR proteins of Gram-positive bacteria used in the study are tabulated in Supplementary Table S12.

### Amino Acid composition

The fraction of each amino acid for the Gram-positive LuxI and LuxR containing proteins was calculated and compared with Gram-negative bacteria to obtain the distinctiveness (predominance and depletion of residues) among them^22, 55, 56^. Amino Acid Composition was calculated using programs built in Perl scripting language. The formula for calculating AAC is:

$$Comp(x)=\frac{{A}_{x}}{N}$$

where, *Comp (x)* is the composition of amino acid (*x)*; *A*_*x*_ is number of the residues of type *x* and *N* is total residues in protein. In this study, amino acids with fold changes ≤0.80 or ≥1.20 and *p-value* < 0.05 are considered significant^57^.

### Motif scanning

The motif is a conserved pattern of amino acid with a specific function. Despite extracting continuous motif, we extracted gapped motif using GLAM2 *v*1056 (Gapped Local Alignment of Motifs) software^58^ in putative LuxI/LuxR proteins of Gram-positive bacteria. Furthermore, the scanning of extracted motif in a sequence database (LuxI/LuxR of Gram-negative bacteria) was done using GLAM2SCAN *v*1056 software. The high intensity of the GLAM2 score for particular motif indicates its strength.

### Domain analysis

The domain is a conserved portion of a protein sequence (and/or structure) that can evolve, function and exist independently from rest protein. For extensive searching of the domain from proteins we used two repositories: i) InterPro ii) NCBI-Conserved Domain Database (CDD) with hit type “*specific*” due to slight variations in domains among them. Moreover, domain analysis was done in two ways for both the strategies i.e. occurrence of domain individually and as combination per protein.

### Gene ontology

Functional annotations of LuxI/LuxR proteins were done using Gene Ontology^59^, on the basis of three domains namely biological process, molecular function and cellular component. “*Biological process*” determines pathways or processes formed by activities of gene product; “*molecular function*” shows the molecular activities of gene products and “*cellular component*” gave the subcellular location of gene product. We extracted the information of preferred GO functions assigned to protein sequences and depicted them in the form of bubble charts in R using ggplot2 library.

### Ligand-binding prediction

To get the insight of the specificity of the LuxR sequences of Gram-positive bacteria towards the ligands, their prediction for ligand-binding potential was performed by COACH^60^ software available in I-TASSER package. It identifies the ligands using binding-specific substructure comparison (TM-SITE) and sequence profile alignment (S-SITE) approach.

### Clustering

Cluster analysis was done through CLANS (Cluster Analysis of Sequences) software^61^. It is a java application based on Fruchterman-Reingold graph layout algorithm for protein families visualization. CLANS perform BLAST/PSIBLAST searches for each sequence using “*all-against-all*” approach for calculating pair-wise attraction values as high scoring segment pair’s *p*-*values*. This analysis was performed to evaluate taxonomic relatedness among species of the LuxI and LuxR sequences of Gram-positive bacteria.

### Multiple Sequence Alignment

Both LuxI and LuxR containing sequences were aligned using MAFFT software^62^ against *V*. *fischeri* LuxI (P12747) and LuxR (P12746) respectively. It is a similarity-based method built employing fast Fourier transform algorithm for identifying the homologous region of the sequences by translating amino acids to their respective volume and polarity values. Further, the aligned output was visualized through Jalview^63^ alignment viewer software to extract consensus information.

### Phylogenetic analyses

Reconstruction of Gram-positive bacteria putative LuxI/LuxR containing protein sequences was done to establish the evolutionary history along respective sequences from BLAST similarity hits using Molecular Evolutionary Genetics Analysis (MEGA) 7.0 package^19, 64–66^. All the sequences were first aligned using MUSCLE tool^67^ integrated into MEGA 7.0. Further, “*best protein model*” algorithm of MEGA 7.0 was exploited to identify the most preferred model for tree building *via* Maximum-likelihood method.

For LuxI containing protein, Maximum-likelihood (ML) tree building was employed on sequences from Gram-positive bacteria (11), respective BLAST hits (Supplementary Table S13). ML tree was built using Le Gascuel (LG) model^68^ using a discrete gamma distribution (+G) to establish evolutionary rates among sites along with rate variation measurement allowed for some sites to be evolutionary invariable (+I). Moreover, LuxR containing proteins’ evolutionary history was inferred using Gram-positive bacteria (70) and their respective BLAST hits (Supplementary Table S13). ML tree reconstruction was completed using LG + G method. Statistical support for all the tree reconstruction was computed by bootstrap analysis using 1000 pseudo-replicates. Moreover, the 16s rRNA gene tree of Gram-positive bacteria and Gram-negative bacteria used in the study is provided in Supplementary Figure S5.

## Electronic supplementary material

Supplementary Information
