# Supplementary material for: In silico analyses of conservational, functional and phylogenetic distribution of the LuxI and LuxR homologs in Gram-positive bacteria
Source: Sci Rep. 2017 Aug 1;7:6969. doi: 10.1038/s41598-017-07241-5 (PMC5539150; doi:10.1038/s41598-017-07241-5)
Supplement: Supplementary file 1 — Supplementary Information [file 41598_2017_7241_MOESM1_ESM.pdf]

In silico analysis of conservational, functional and phylogenetic distribution of the LuxI and LuxR homologs in Gram-positive bacteria

Akanksha Rajput and Manoj Kumar*

Bioinformatics Centre, Institute of Microbial Technology, Council of Scientific and Industrial Research, Sector 39A, Chandigarh-160036, India

* To whom correspondence should be addressed: manojk@imtech.res.in

Akanksha Rajput

Bioinformatics Centre, Institute of Microbial Technology, Council of Scientific and Industrial Research, Sector 39A, Chandigarh-160036, India

Email: akanksha@imtech.res.in

Manoj Kumar

Bioinformatics Centre, Institute of Microbial Technology, Council of Scientific and Industrial Research, Sector 39A, Chandigarh-160036, India

Email: manojk@imtech.res.in

Supplementary Information

Supplementary Figure S1. Bar graph showing amino acid composition. Comparison between Gram-positive and Gram-negative bacteria **(a)** LuxI containing protein, **(b)** LuxR containing proteins.

Supplementary Figure S2. Bubble plot showing distribution of Gene Ontology (GO) annotations (a) 09 biological processes *(details of GO IDs in Supplementary Table S8)*, **(b) of 19 molecular functions** *(details of GO IDs in Supplementary Table S8)*.

Supplementary Figure S3. Clustering of QS regulators using CLANS software at different *p-values*. (a) 11 LuxI containing Gram-positive sequences [at *p-value* 1e-20], (b) 800 LuxR containing Gram-positive sequences using CLAN software [*p-value* 1e-45].

Supplementary Figure S4. Phylogenetic tree reconstruction using Maximum Likelihood method for Gram-positive bacteria and their respective Gram-negative BLAST hits (a) LuxI containing sequences, (b) LuxR containing sequences, and [*Gram-positive bacteria (●), and Gram-negative bacteria (■)*].

Supplementary Figure S5. Reconstruction of 16s rRNA gene tree of Gram-positive, and Gram-negative used in the study employing Maximum likelihood method with Kimura-2-parameter model at 1000 bootstrap support. [*Gram-positive bacteria (●) and Gram-negative bacteria (■)*].

Supplementary Table S1. List of top-10 LuxI containing Gram-positive bacteria sequence motifs extracted using GLAM2 software with information of Web logo, total alignment score, motif width and sequence coverage.

Supplementary Table S2. List of top-10 LuxR containing Gram-positive bacteria sequence motifs extracted using GLAM2 software with information of Web logo, total alignment score, motif width and sequence coverage.

Supplementary Table S3. Table showing 78 unique domains extracted from Interpro from LuxR containing Gram-positive bacteria.

Supplementary Table S4. Table showing domain definition of all 78 unique domains extracted from InterPro database from LuxR containing proteins along with their homology. *[ABD, Autoinducer Binding Domain; DNAB, DNA Binding Domain; NA , Not Available]*

Supplementary Table S5. Table showing 85 domain combinations extracted from Interpro from LuxR containing Gram-positive bacteria.

Supplementary Table S6. Table showing 70 unique domains extracted from NCBI-CDD from LuxR containing Gram-positive bacteria.

Supplementary Table S7. Table showing 162 domain combinations extracted from NCBI CDD from LuxR containing Gram-positive bacteria.

Supplementary Table S8. Table showing details of GO IDs involved in biological processes and molecular functions.

Supplementary Table S9. List of ligands that bind to putative LuxR proteins of Gram-positive bacteria extracted using COACH software available in I-TASSER package.

Supplementary Table S10. List of all the conserved residues in 11 LuxI containing Gram-positive bacteria sequences against *V. fischeri* LuxI sequence extracted using MAFFT alignment tool and viewed using Jalview software.

Supplementary Table S11. List of all the conserved residues in 800 LuxR containing Gram-positive bacteria sequences against *V. fischeri* LuxR sequence extracted using MAFFT alignment tool and viewed using Jalview software.

Supplementary Table S12. List of the LuxI and LuxR proteins of Gram-positive bacteria used in amino acid composition, motif, domain, gene ontology and clustering analyses.

Supplementary Table S13. List of the LuxI and LuxR containing proteins of Gram-positive bacteria, and Gram-negative bacteria used in phylogenetic analyses.

Supplementary Figures

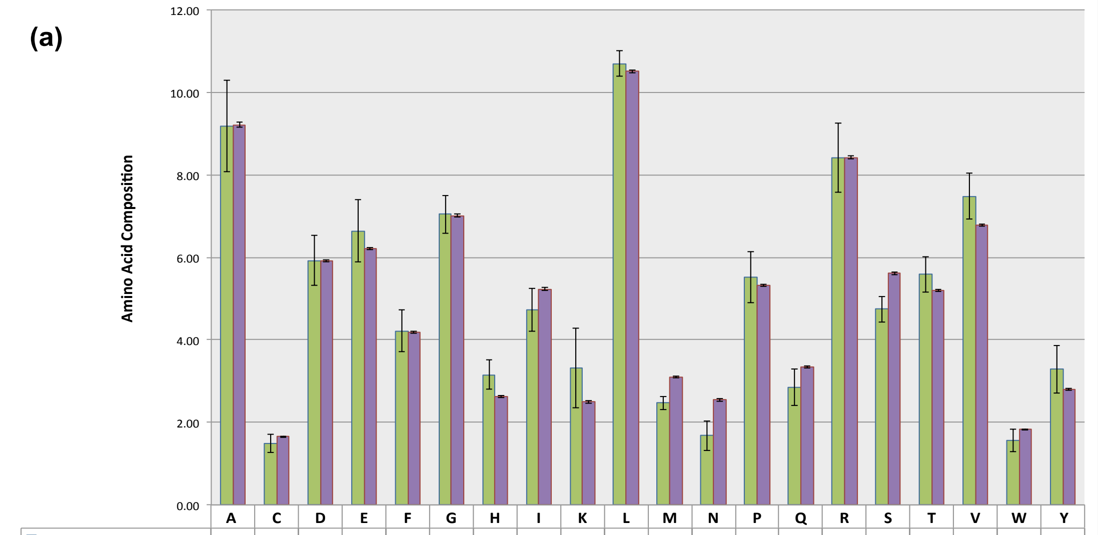

	A	C	D	E	F	G	H	I	K	L	M	N	P	Q	R	S	T	V	W	Y
Gram-positive bacteria	9.18	1.49	5.92	6.64	4.22	7.04	3.16	4.73	3.32	10.70	2.47	1.68	5.51	2.85	8.41	4.74	5.59	7.48	1.56	3.29
Gram-negative bacteria	9.22	1.65	5.92	6.21	4.18	7.01	2.62	5.23	2.50	10.51	3.10	2.54	5.32	3.34	8.43	5.62	5.20	6.78	1.83	2.80

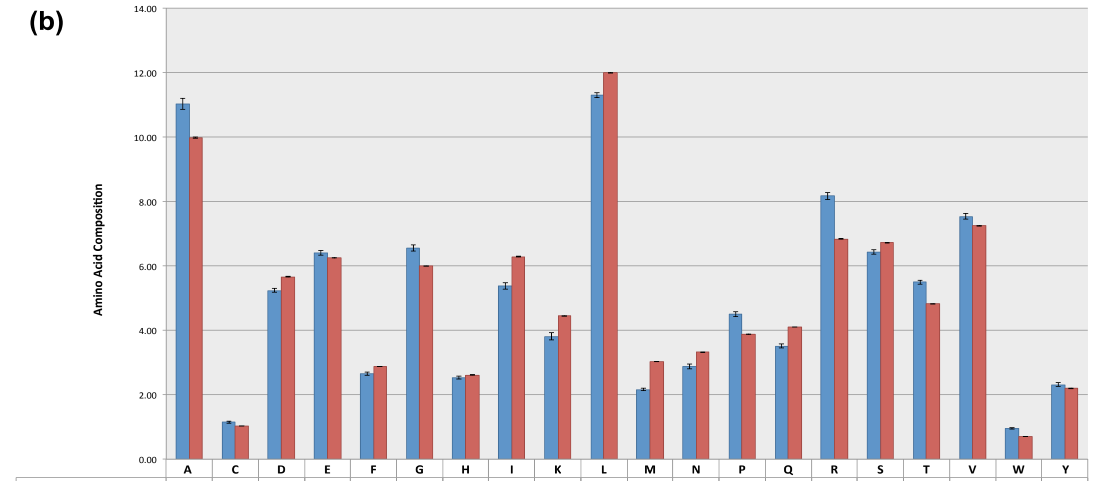

	A	C	D	E	F	G	H	I	K	L	M	N	P	Q	R	S	T	V	W	Y
Gram-positive bacteria	11.03	1.15	5.24	6.40	2.65	6.55	2.53	5.38	3.81	11.30	2.16	2.88	4.50	3.51	8.17	6.43	5.49	7.53	0.96	2.32
Gram-negative bacteria	9.98	1.04	5.66	6.25	2.88	6.00	2.61	6.29	4.45	11.99	3.03	3.32	3.88	4.11	6.84	6.72	4.82	7.24	0.70	2.19

Supplementary Figure S1. Bar graph showing amino acid composition. Comparison between Gram-positive and Gram-negative bacteria (a) LuxI containing protein, (b) LuxR containing proteins.

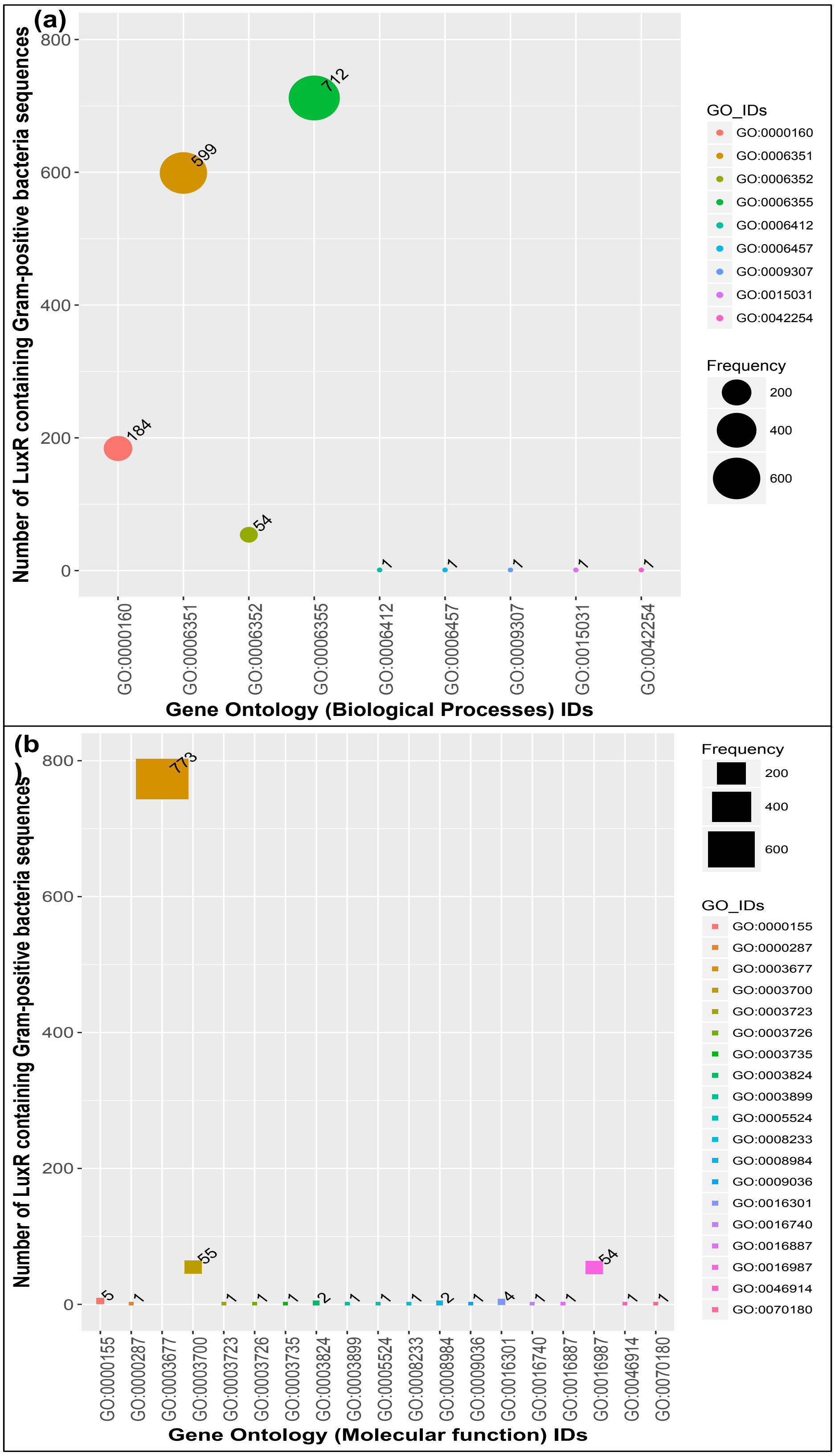

Supplementary Figure S2. Bubble plot showing distribution of Gene Ontology (GO) annotations (a) 09 biological processes *(details of GO IDs in Supplementary Table S8)*, **(b) of 19 molecular functions** *(details of GO IDs in Supplementary Table S8)*.

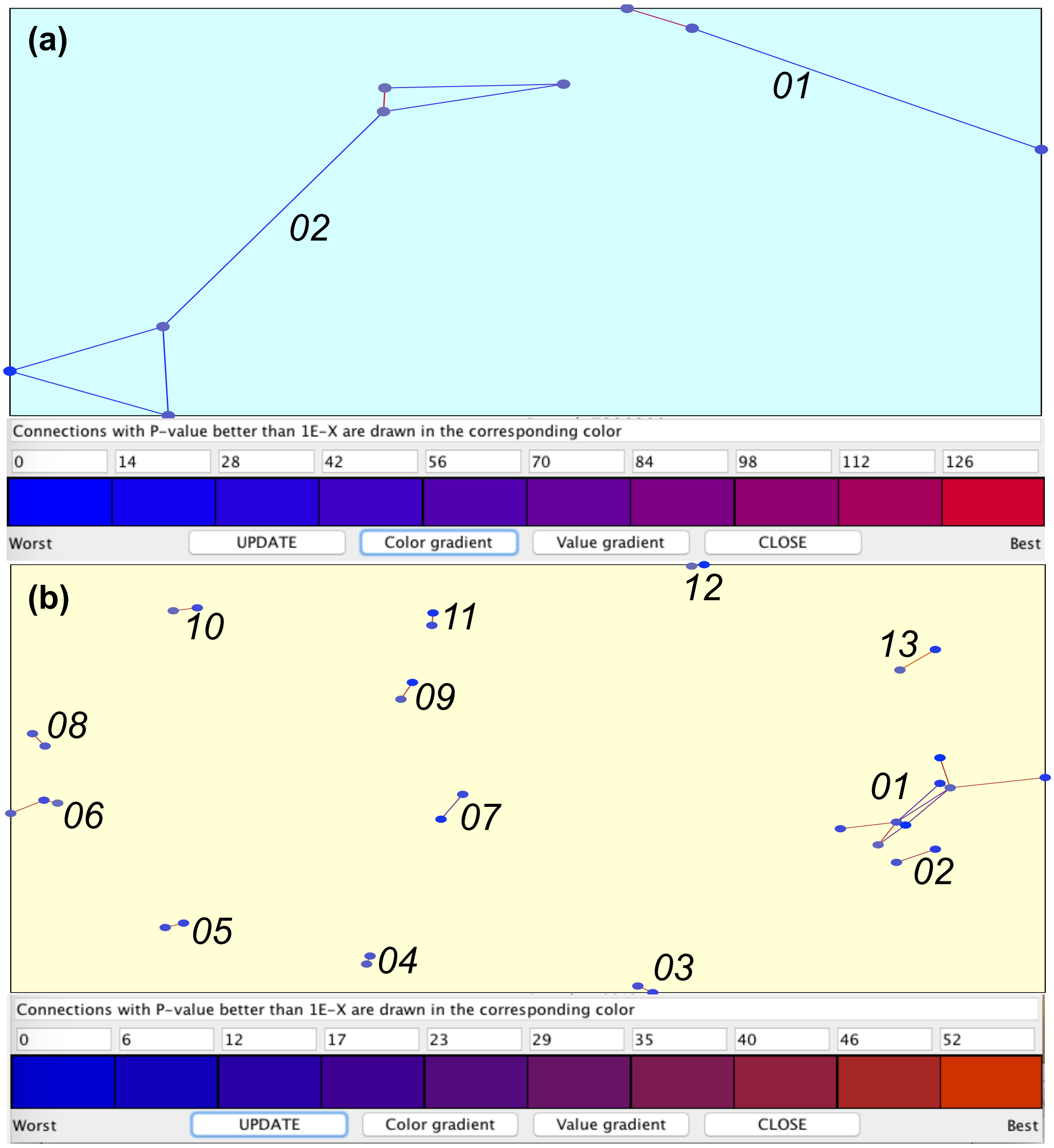

Supplementary Figure S3. Clustering of QS regulators using CLANS software at different *p-values*. (a) 11 LuxI containing Gram-positive sequences [at *p-value* 1e-20], (b) 800 LuxR containing Gram-positive sequences using CLAN software [*p-value* 1e-45].

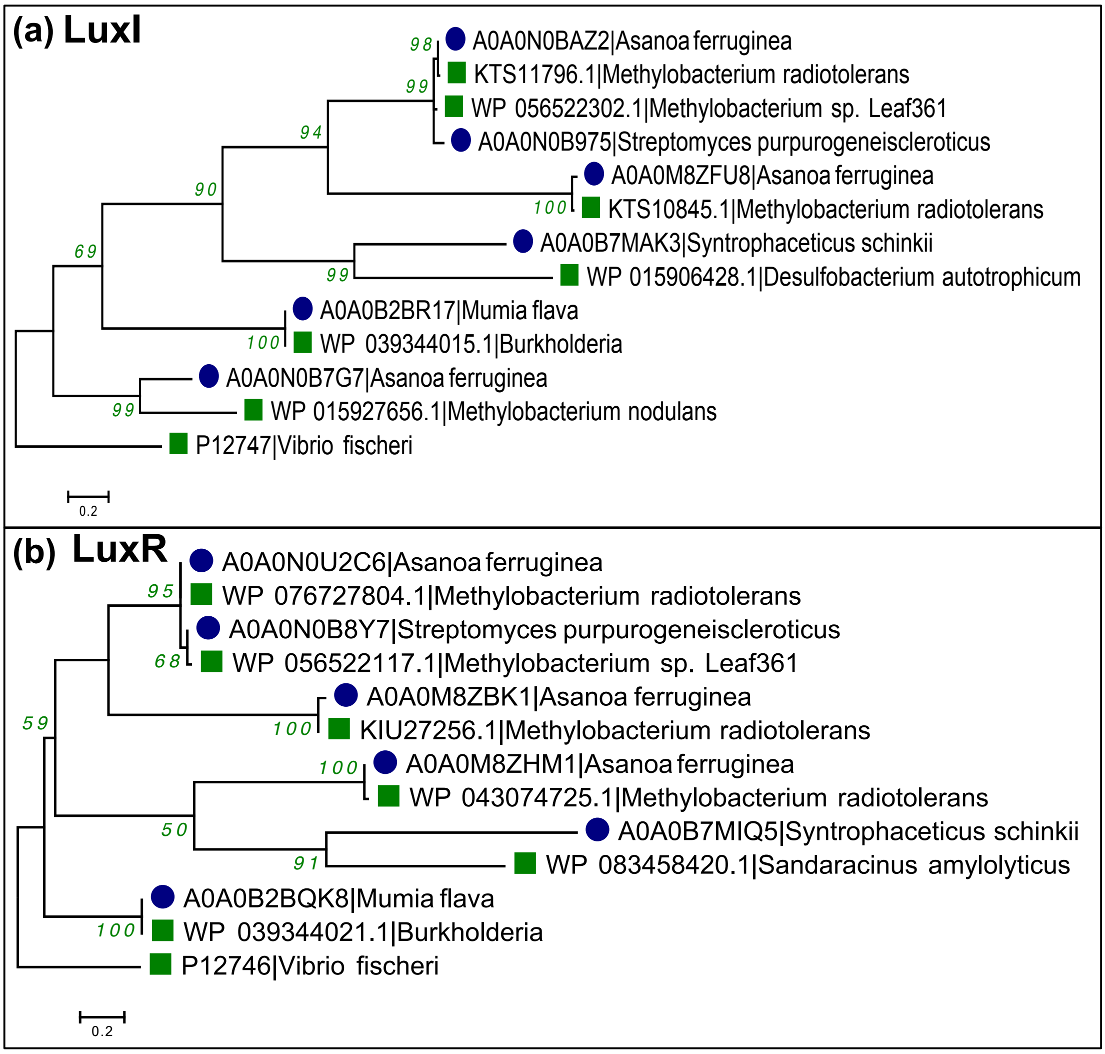

Supplementary Figure S4. Phylogenetic tree reconstruction using Maximum Likelihood method for Gram-positive bacteria and their respective Gram-negative BLAST hits (a) LuxI containing sequences, (b) LuxR containing sequences, and [*Gram-positive bacteria* (●), and *Gram-negative bacteria* (■)].

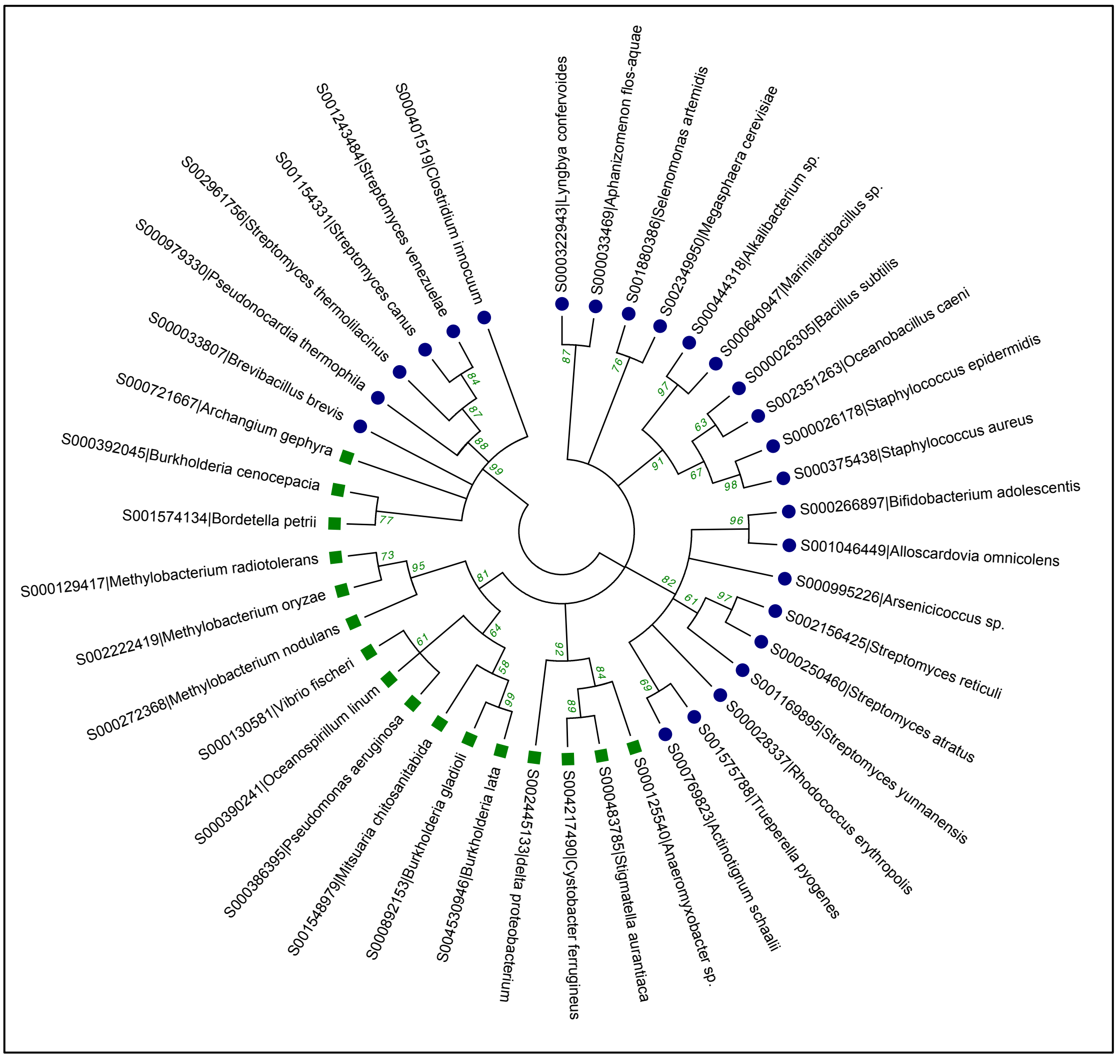

Supplementary Figure S5. Reconstruction of 16s rRNA gene tree of Gram-positive, and Gram-negative used in the study employing Maximum likelihood method with Kimura-2-parameter model at 1000 bootstrap support. [*Gram-positive bacteria* (●) *and Gram-negative bacteria* (■)].

Supplementary Tables

Supplementary Table S1. List of top-10 LuxI containing Gram-positive bacteria sequence motifs extracted using GLAM2 software with information of Web logo, total alignment score, motif width and sequence coverage.

S.No	Weblogo	Total alignment score	Motif Width	Percentage coverage (11)
Motif 1	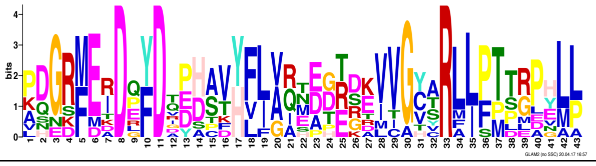	387.12	43	10
Motif 2	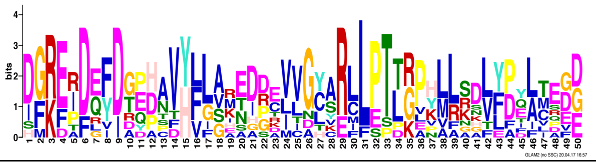	367.07	50	10
Motif 3	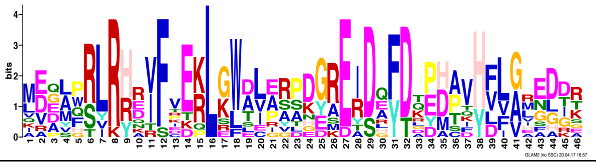	353.04	46	11
Motif 4	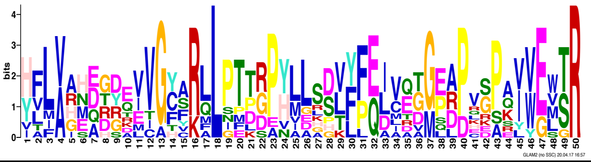	335.26	50	10
Motif 5	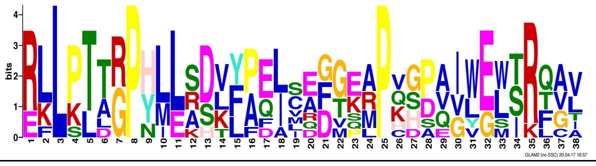	263.77	38	08
Motif 6	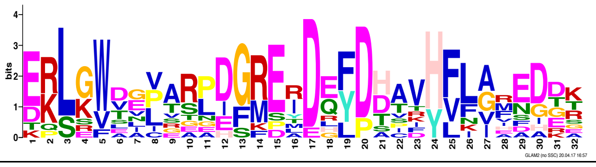	222.88	32	11
Motif 7	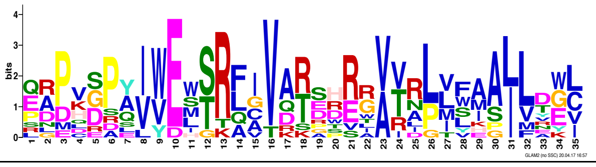	204.02	35	11
Motif 8	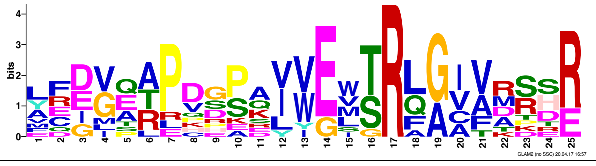	179.97	25	11

Motif 9	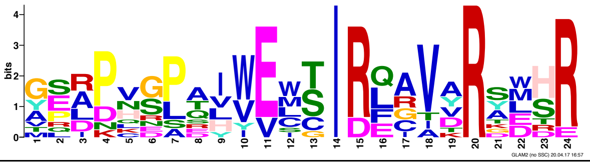	149.30	24	11
Motif 10	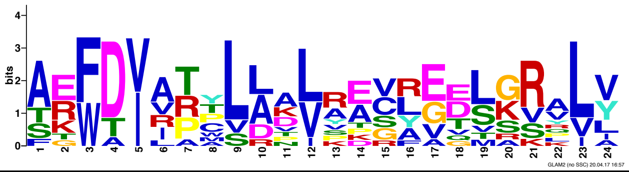	70.16	24	11

Supplementary Table S2. List of top-10 LuxR containing Gram-positive bacteria sequence motifs extracted using GLAM2 software with information of Web logo, total alignment score, motif width and sequence coverage.

S.No	Weblogo	Total alignment score	Motif Width	Percentage coverage (800)
Motif 1	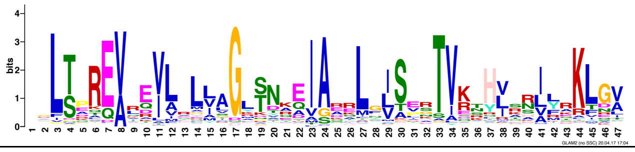	50338.8	47	799
Motif 2	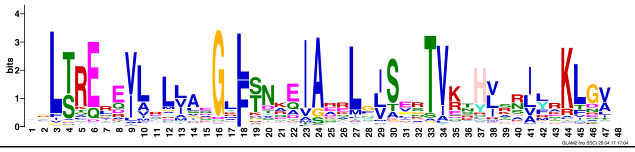	50019.4	48	799
Motif 3	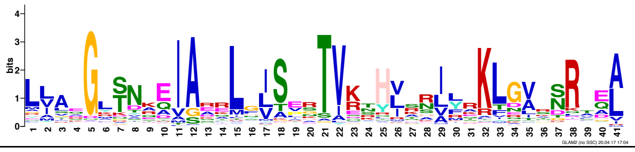	44093.1	41	798
Motif 4	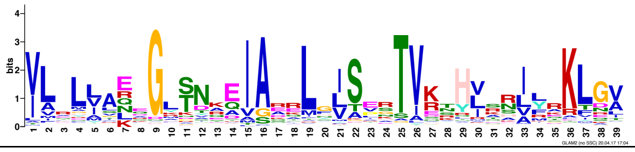	42663.5	39	799
Motif 5	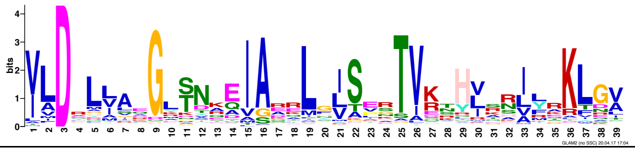	42656.4	39	799
Motif 6	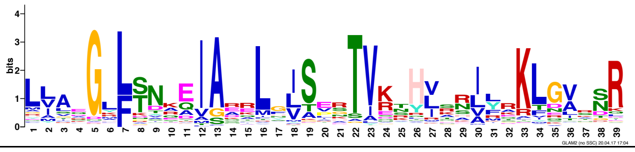	42262.4	39	798
Motif 7	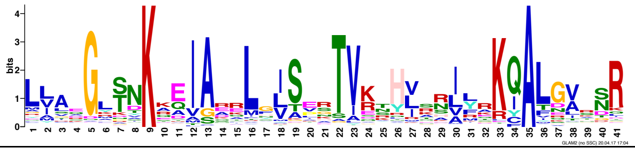	42015.4	41	798

Motif 8	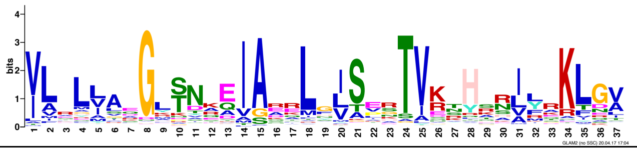	40116.9	37	799
Motif 9	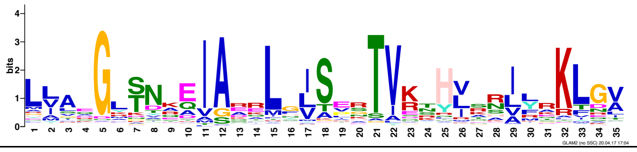	39421.0	35	798
Motif 10	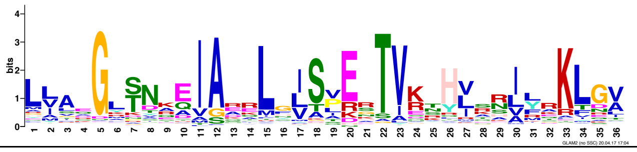	38610.1	36	798

Supplementary Table S3. Table showing 78 unique domains extracted from Interpro from LuxR containing Gram-positive bacteria.

InterPro ID	Frequency
IPR000792	800
IPR011991	792
IPR016032	754
IPR011006	233
IPR001789	194
IPR029016	45
IPR013325	42
IPR014284	42
IPR011990	41
IPR007627	36
IPR013324	33
IPR013249	26
IPR005143	18
IPR000014	17
IPR009057	9
IPR003018	8
IPR013656	6
IPR007630	5
IPR027417	5
IPR000700	4
IPR018771	4
IPR000253	3
IPR000943	3
IPR003594	3
IPR008984	3
IPR010982	3
IPR013767	3
IPR014325	3
IPR001610	2
IPR013655	2
IPR013785	2
IPR016040	2
IPR018656	2
IPR019092	2
IPR029787	2
IPR000073	1

IPR000607	1
IPR000835	1
IPR001790	1
IPR002363	1
IPR003439	1
IPR003482	1
IPR003593	1
IPR004360	1
IPR005119	1
IPR005545	1
IPR006674	1
IPR007624	1
IPR008523	1
IPR008881	1
IPR009081	1
IPR009959	1
IPR011008	1
IPR011051	1
IPR011659	1
IPR011712	1
IPR014218	1
IPR014322	1
IPR014710	1
IPR015421	1
IPR015813	1
IPR015943	1
IPR019071	1
IPR019848	1
IPR019885	1
IPR020542	1
IPR020846	1
IPR021235	1
IPR022689	1
IPR022742	1
IPR022973	1
IPR023187	1
IPR025201	1
IPR025847	1
IPR029058	1
IPR029068	1
IPR029149	1
IPR032710	1

Supplementary Table S4. Table showing domain definition of all 78 unique domains extracted from InterPro database from LuxR containing proteins along with their homology. *[ABD, Autoinducer Binding Domain; DNAB, DNA Binding Domain; NA , Not Available]*

Domain	Frequency	Homology	Definition
IPR000792	800	DNAB	Transcription factor LuxR-like, autoinducer-binding domain
IPR011991	792	DNAB	Winged helix-turn-helix DNA-binding domain
IPR016032	754	DNAB	Signal transduction response regulator, C-terminal effector
IPR011006	233	ABD	CheY-like superfamily
IPR001789	194	ABD	Signal transduction response regulator, receiver domain
IPR029016	45	ABD	GAF domain-like
IPR013325	42	DNAB	RNA polymerase sigma factor, region 2
IPR014284	42	DNAB	RNA polymerase sigma-70 like domain

IPR011990	41	ABD	Tetratricopeptide-like helical domain
IPR007627	36	DNAB	RNA polymerase sigma-70 region 2
IPR013324	33	DNAB	RNA polymerase sigma factor, region 3/4
IPR013249	26	DNAB	RNA polymerase sigma factor 70, region 4 type 2
IPR005143	18	ABD	Transcription factor LuxR-like, autoinducer-binding domain
IPR000014	17	ABD	PAS domain
IPR009057	9	DNAB	Homeobox domain-like
IPR003018	8	ABD	GAF domain
IPR013656	6	ABD	PAS fold-4
IPR007630	5	DNAB	RNA polymerase sigma-70 region 4
IPR027417	5	NA	P-loop containing nucleoside triphosphate hydrolase
IPR018771	4	ABD	PocR domain
IPR000700	4	NA	PAS-associated, C-terminal
IPR000253	3	ABD	Forkhead-associated (FHA) domain
IPR008984	3	ABD	SMAD/FHA domain
IPR013767	3	ABD	PAS fold
IPR000943	3	DNAB	RNA polymerase sigma-70
IPR010982	3	DNAB	Lambda repressor-like, DNA-binding domain
IPR003594	3	NA	Histidine kinase-like ATPase, C-terminal domain
IPR014325	3	NA	RNA polymerase sigma-E factor, actinobacteria
IPR016040	2	ABD	NAD(P)-binding domain
IPR001610	2	NA	PAC motif
IPR013655	2	NA	PAS fold-3
IPR013785	2	NA	Aldolase-type TIM barrel
IPR018656	2	NA	Domain of unknown function DUF2087
IPR019092	2	NA	CRISPR-assoc protein, NE0113/Csx13
IPR029787	2	NA	Nucleotide cyclase
IPR005119	1	ABD	LysR, substrate-binding
IPR009081	1	ABD	Phosphopantetheine binding ACP domain
IPR025201	1	ABD	Sensor protein KdpD, transmembrane domain
IPR025847	1	ABD	MEDS domain
IPR000835	1	DNAB	MarR-type HTH domain
IPR007624	1	DNAB	RNA polymerase sigma-70 region 3
IPR019885	1	DNAB	Transcription regulator HTH, AsnC-type, conserved site
IPR023187	1	DNAB	Transcriptional regulator MarR-type, conserved site
IPR000073	1	NA	Alpha/beta hydrolase fold-1
IPR000607	1	NA	Double-stranded RNA-specific adenosine deaminase
IPR001790	1	NA	Ribosomal protein L10P
IPR002363	1	NA	Ribosomal protein L10, eubacterial, conserved site
IPR003439	1	NA	ABC transporter-like
IPR003482	1	NA	Transcription factor WhiB
IPR003593	1	NA	AAA+ ATPase domain
IPR004360	1	NA	Glyoxalase/fosfomycin resistance/dioxygenase domain
IPR005545	1	NA	YCII-related
IPR006674	1	NA	HD domain
IPR008523	1	NA	Protein of unknown function DUF805
IPR008881	1	NA	Trigger factor, ribosome-binding, bacterial
IPR009959	1	NA	Polyketide cyclase SnoaL-like domain
IPR011008	1	NA	Dimeric alpha-beta barrel
IPR011051	1	NA	RmlC-like cupin domain
IPR011659	1	NA	WD40-like Beta Propeller
IPR011712	1	NA	Signal transduction histidine kinase, subgroup 3, dimerisation and phosphoacceptor domain
IPR014218	1	NA	RNA polymerase sigma-H type
IPR014322	1	NA	RNA polymerase sigma-B/F/G type
IPR014710	1	NA	RmlC-like jelly roll fold
IPR015421	1	NA	Pyridoxal phosphate-dependent transferase, major region,

			subdomain 1
IPR015813	1	NA	Pyruvate/Phosphoenolpyruvate kinase-like domain
IPR015943	1	NA	WD40/YVTN repeat-like-containing domain
IPR019071	1	NA	Restriction endonuclease, type II, XcyI
IPR019848	1	NA	CRISPR-associated protein, Csx14
IPR020542	1	NA	Aspartate carbamoyltransferase regulatory subunit, C-terminal
IPR020846	1	NA	Major facilitator superfamily domain
IPR021235	1	NA	Protein of unknown function DUF2637
IPR022689	1	NA	Iron dependent repressor
IPR022742	1	NA	Serine aminopeptidase, S33
IPR022973	1	NA	Ribosomal protein L10
IPR029058	1	NA	Alpha/Beta hydrolase fold
IPR029068	1	NA	Glyoxalase/Bleomycin resistance protein/Dihydroxybiphenyl dioxygenase
IPR029149	1	NA	Creatinase/Aminopeptidase P/Spt16, N-terminal
IPR032710	1	NA	NTF2-like domain

Supplementary Table S5. Table showing 85 domain combinations extracted from Interpro from LuxR containing Gram-positive bacteria**.**

**InterPro ID**	**Frequency**
IPR016032+IPR000792+IPR011991	339
IPR011006+IPR016032+IPR001789+IPR000792+IPR011991	179
IPR016032+IPR011990+IPR000792+IPR011991	41
IPR011006+IPR016032+IPR000792+IPR011991	40
IPR029016+IPR016032+IPR000792+IPR011991	36
IPR016032+IPR005143+IPR000792+IPR011991	17
IPR014284+IPR007627+IPR013249+IPR013325+IPR013324+IPR000792+IPR011991	10
IPR014284+IPR007627+IPR013325+IPR016032+IPR000792+IPR011991	10
IPR003018+IPR029016+IPR016032+IPR000792+IPR011991	8
IPR011006+IPR001789+IPR000792+IPR011991	7
IPR000014+IPR016032+IPR000792+IPR011991	6
IPR014284+IPR007627+IPR013325+IPR013324+IPR000792+IPR011991	5
IPR000014+IPR013656+IPR016032+IPR000792+IPR011991	4
IPR013249+IPR016032+IPR000792+IPR011991	4
IPR018771+IPR016032+IPR000792+IPR011991	4
IPR027417+IPR016032+IPR000792+IPR011991	4
IPR000253+IPR016032+IPR008984+IPR000792+IPR011991	3
IPR000792+IPR011991	3
IPR000014+IPR013767+IPR016032+IPR000792+IPR011991	2
IPR007627+IPR013325+IPR016032+IPR000792+IPR011991	2
IPR007630+IPR013324+IPR000792+IPR011991	2
IPR011006+IPR013249+IPR016032+IPR001789+IPR000792+IPR011991	2
IPR013785+IPR016032+IPR000792+IPR011991	2
IPR014284+IPR007627+IPR007630+IPR013325+IPR013324+IPR000792+IPR011991	2
IPR014284+IPR013249+IPR013325+IPR013324+IPR000792+IPR011991	2
IPR014284+IPR013325+IPR016032+IPR000792+IPR011991	2
IPR014284+IPR014325+IPR007627+IPR013249+IPR013325+IPR013324+IPR000792+IPR011991	2
IPR016032+IPR000792	2
IPR016032+IPR001789+IPR000792+IPR011991	2
IPR016040+IPR016032+IPR000792+IPR011991	2
IPR029787+IPR016032+IPR000792+IPR011991	2
IPR000014+IPR000700+IPR013656+IPR016032+IPR000792+IPR011991	1
IPR000014+IPR000700+IPR013767+IPR016032+IPR000792+IPR011991	1
IPR000014+IPR013655+IPR016032+IPR000792+IPR011991	1

IPR000607+IPR016032+IPR000792+IPR011991	1
IPR000835+IPR022689+IPR000792+IPR023187+IPR011991	1
IPR001610+IPR000014+IPR000700+IPR013656+IPR016032+IPR000792+IPR011991	1
IPR001610+IPR000014+IPR000700+IPR016032+IPR000792+IPR011991	1
IPR003593+IPR003439+IPR011006+IPR027417+IPR016032+IPR001789+IPR000792+IPR011991	1
IPR003594+IPR011712+IPR016032+IPR000792+IPR011991	1
IPR003594+IPR016032+IPR000792+IPR011991	1
IPR005119+IPR016032+IPR000792+IPR011991	1
IPR006674+IPR016032+IPR000792+IPR011991	1
IPR008523+IPR016032+IPR000792+IPR011991	1
IPR009057+IPR000792+IPR011991	1
IPR009057+IPR010982+IPR014284+IPR013249+IPR013324+IPR000792+IPR011991	1
IPR009057+IPR010982+IPR019885+IPR000792+IPR011991	1
IPR009057+IPR014284+IPR000943+IPR007627+IPR013325+IPR013324+IPR000792	1
IPR009057+IPR014284+IPR013325+IPR016032+IPR000792	1
IPR009057+IPR016032+IPR000792	1
IPR009057+IPR016032+IPR000792+IPR011991	1
IPR009081+IPR014284+IPR007627+IPR013325+IPR016032+IPR000792+IPR011991	1
IPR009959+IPR032710+IPR016032+IPR000792+IPR011991	1
IPR010982+IPR016032+IPR000792+IPR011991	1
IPR011006+IPR003594+IPR001789+IPR000792+IPR011991	1
IPR011006+IPR009057+IPR016032+IPR001789+IPR000792	1
IPR011006+IPR015813+IPR016032+IPR000792+IPR011991	1
IPR011006+IPR016032+IPR001789+IPR008881+IPR000792+IPR011991	1
IPR011008+IPR016032+IPR000792+IPR011991+IPR005545	1
IPR011659+IPR016032+IPR000792+IPR015943+IPR011991	1
IPR013249+IPR013324+IPR016032+IPR000792+IPR011991	1
IPR013324+IPR000792+IPR011991	1
IPR013324+IPR016032+IPR000792+IPR011991	1
IPR013655+IPR016032+IPR000792	1
IPR014284+IPR013249+IPR013324+IPR000792+IPR011991	1
IPR014284+IPR013325+IPR013324+IPR000792+IPR011991	1
IPR014284+IPR014218+IPR007627+IPR013249+IPR013325+IPR013324+IPR000792+IPR011991	1
IPR014284+IPR014322+IPR000943+IPR007627+IPR007624+IPR007630+IPR013325+IPR013324+IPR000792+IPR011991	1
IPR014284+IPR014325+IPR000943+IPR007627+IPR013249+IPR013325+IPR013324+IPR000792+IPR011991	1
IPR014710+IPR011051+IPR016032+IPR000792+IPR011991	1
IPR015421+IPR016032+IPR000792+IPR011991	1
IPR016032+IPR000792+IPR003482+IPR011991	1
IPR018656+IPR016032+IPR000792+IPR011991	1
IPR019071+IPR016032+IPR000792+IPR011991	1
IPR019092+IPR016032+IPR000792+IPR011991	1
IPR019848+IPR019092+IPR016032+IPR000792+IPR011991	1
IPR020542+IPR018656+IPR016032+IPR000792+IPR011991	1
IPR020846+IPR016032+IPR000792+IPR011991	1
IPR021235+IPR009057+IPR000792	1
IPR025201+IPR016032+IPR000792+IPR011991	1
IPR025847+IPR016032+IPR000792+IPR011991	1
IPR029016+IPR016032+IPR005143+IPR000792+IPR011991	1
IPR029058+IPR000073+IPR022742+IPR016032+IPR000792+IPR011991	1
IPR029068+IPR004360+IPR016032+IPR000792+IPR011991	1
IPR029149+IPR022973+IPR002363+IPR001790+IPR013249+IPR016032+IPR000792+IPR011991	1

Supplementary Table S6. Table showing 70 unique domains extracted from NCBI-CDD from LuxR containing Gram-positive bacteria.

NCBI Domains	Frequency
CitB	668
HTH_LUXR	646
LuxR_C_like	532
GerE	266
REC	99
sigma70-ECF	95
CsgD	88
PAS	34
Response_reg	28
Sigma70_r2	21
RpoE	20
Sigma70_r4	18
Sigma70_r4_2	18
FHA	11
FhlA	6
GAF	6
Autoind_bind	5
HATPase_c	5
PAS_9	5
FliA	4
GAF_2	4
HTH_24	3
PAS_3	3
SigE-fam_strep	3
AAA_16	2
Cas_NE0113	2
Csx1_III-U	2
DUF2087	2
FixJ	2
HDc	2
Lrp	2
MarR	2
PAC	2
Ribosomal_L10	2
CheY	1
Cls	1
Cupin_2	1
DUF2637	1
DUF4118	1
DUF805	1
GAF_3	1
GGDEF	1
Glyoxalase	1
HD_5	1
HisKA_3	1
HTH_23	1
HTH_38	1
HTH_ARSR	1
HTH_MARR	1
LysR	1
LysR_substrate	1
MEDS	1
MhpC	1

OmpR	1
PAS_4	1
PAS_8	1
PBP2_LTTR_substrate	1
PD40	1
PKS_PP	1
PRK08295	1
PRK09639	1
PRK09652	1
RplJ	1
rplJ	1
sensory_box	1
Tig	1
TolB	1
Trigger_N	1
Whib	1
yhaH	1

Supplementary Table S7. Table showing 162 domain combinations extracted from Interpro from LuxR containing Gram-positive bacteria.

**Domain combinations**	**Frequency**
CitB + HTH_LUXR + LuxR_C_like + GerE	106
CitB + HTH_LUXR + LuxR_C_like	92
CitB	48
HTH_LUXR + CitB + LuxR_C_like	48
CitB + HTH_LUXR + GerE + LuxR_C_like	47
HTH_LUXR + CitB + LuxR_C_like + GerE	36
HTH_LUXR + LuxR_C_like + CitB	31
CitB + REC + HTH_LUXR	30
CitB + REC + Response_reg + HTH_LUXR	17
HTH_LUXR + CitB + GerE + LuxR_C_like	16
CitB + HTH_LUXR	15
HTH_LUXR + LuxR_C_like + CsgD	13
CitB + HTH_LUXR + LuxR_C_like + sigma70-ECF	11
HTH_LUXR + CitB	11
CitB + REC	10
CsgD	9
HTH_LUXR + CitB + LuxR_C_like + sigma70-ECF	8
CsgD + HTH_LUXR + LuxR_C_like	7
sigma70-ECF	7
CitB + HTH_LUXR + REC	6
CitB + REC + REC	6
CitB + Response_reg + REC + HTH_LUXR	6
HTH_LUXR + CitB + LuxR_C_like + CsgD	6
HTH_LUXR + CsgD	6
Sigma70_r2	5
sigma70-ECF + Sigma70_r2	5
HTH_LUXR + CsgD + LuxR_C_like	4
HTH_LUXR + GerE + CitB + LuxR_C_like	4
HTH_LUXR + LuxR_C_like + CsgD + sigma70-ECF	4
LuxR_C_like + HTH_LUXR + CitB	4
CitB + HTH_LUXR + GerE + LuxR_C_like + sigma70-ECF	3
CitB + HTH_LUXR + REC + GerE	3
CitB + LuxR_C_like + HTH_LUXR	3
CitB + REC + HTH_LUXR + sigma70-ECF	3
CitB + sigma70-ECF	3

FliA + Sigma70_r2	3
HTH_LUXR + CitB + LuxR_C_like + GerE + sigma70-ECF	3
HTH_LUXR + GerE + LuxR_C_like + CitB	3
HTH_LUXR + LuxR_C_like + CitB + GerE	3
RpoE + sigma70-ECF + Sigma70_r4 + Sigma70_r4_2	3
SigE-fam_strep + RpoE + Sigma70_r4_2 + Sigma70_r4	3
sigma70-ECF + Sigma70_r4_2	3
Autoind_bind + HTH_LUXR + LuxR_C_like + CsgD	2
Cas_NE0113 + Csx1_III-U + HTH_LUXR + CitB	2
CitB + CsgD	2
CitB + GerE + HTH_LUXR + LuxR_C_like	2
CitB + REC + REC + sigma70-ECF	2
DUF2087 + CitB	2
HTH_LUXR + CitB + LuxR_C_like + GerE + CsgD	2
HTH_LUXR + CsgD + LuxR_C_like + sigma70-ECF	2
HTH_LUXR + LuxR_C_like + CitB + CsgD	2
HTH_LUXR + LuxR_C_like + GerE	2
HTH_LUXR + LuxR_C_like + GerE + CitB	2
HTH_LUXR + LuxR_C_like + GerE + CitB + sigma70-ECF	2
HTH_LUXR + LuxR_C_like + GerE + CsgD	2
RpoE + Sigma70_r2	2
RpoE + sigma70-ECF + Sigma70_r2	2
sigma70-ECF + CitB	2
sigma70-ECF + CsgD	2
Autoind_bind + CsgD + HTH_LUXR + LuxR_C_like	1
Autoind_bind + LuxR_C_like + CsgD + HTH_LUXR	1
Autoind_bind + LuxR_C_like + HTH_LUXR + CsgD	1
CitB + GerE + HTH_LUXR + LuxR_C_like + PAS_3	1
CitB + HTH_23	1
CitB + HTH_LUXR + GerE + LuxR_C_like + FHA + FHA + FHA + FHA	1
CitB + HTH_LUXR + GerE + LuxR_C_like + PAS_9 + PAS + PAS	1
CitB + HTH_LUXR + HD_5 + LuxR_C_like + GerE + HDc + HDc	1
CitB + HTH_LUXR + HTH_LUXR + LuxR_C_like + LuxR_C_like + CsgD	1
CitB + HTH_LUXR + LuxR_C_like + GAF	1
CitB + HTH_LUXR + LuxR_C_like + GAF_2 + GAF	1
CitB + HTH_LUXR + LuxR_C_like + GerE + Cupin_2	1
CitB + HTH_LUXR + LuxR_C_like + GerE + DUF4118	1
CitB + HTH_LUXR + LuxR_C_like + GerE + GAF_2	1
CitB + HTH_LUXR + LuxR_C_like + GerE + HTH_LUXR + LuxR_C_like + CsgD	1
CitB + HTH_LUXR + LuxR_C_like + GerE + HisKA_3	1
CitB + HTH_LUXR + LuxR_C_like + GerE + PAS_3	1
CitB + HTH_LUXR + LuxR_C_like + PAS	1
CitB + HTH_LUXR + LuxR_C_like + PAS + PAS	1
CitB + HTH_LUXR + LuxR_C_like + PAS + PAS_9 + PAS	1
CitB + HTH_LUXR + REC + sigma70-ECF	1
CitB + HTH_LUXR + sigma70-ECF	1
CitB + LuxR_C_like + HTH_LUXR + GerE	1
CitB + LuxR_C_like + HTH_LUXR + GerE + sigma70-ECF	1
CitB + REC + Response_reg + HTH_LUXR + HATPase_c + HATPase_c + HATPase_c	1
CitB + REC + Response_reg + REC	1
CitB + Sigma70_r4_2 + sigma70-ECF	1
CitB + Trigger_N + Tig + HTH_LUXR + LuxR_C_like + GerE	1
CsgD + CitB + sigma70-ECF	1
CsgD + HTH_LUXR + LuxR_C_like + PAS + PAS + PAS_9 + PAS	1
CsgD + HTH_LUXR + PAS_8 + PAS	1
CsgD + PAS + PAS	1

DUF2637 + HTH_38	1
DUF805 + HTH_LUXR + CitB + LuxR_C_like + yhaH + GerE	1
FHA + FHA + CitB + FHA	1
FHA + FHA + FHA + FHA + CitB	1
FixJ + REC + Response_reg + HTH_LUXR	1
FixJ + Response_reg + REC + HTH_LUXR	1
FliA + Sigma70_r2 + Sigma70_r4	1
HTH_LUXR + CitB + CsgD + LuxR_C_like + sigma70-ECF	1
HTH_LUXR + CitB + GerE + LuxR_C_like + FhlA + GAF	1
HTH_LUXR + CitB + GerE + LuxR_C_like + GAF + FhlA	1
HTH_LUXR + CitB + GerE + LuxR_C_like + HATPase_c + HATPase_c	1
HTH_LUXR + CitB + HTH_24 + Lrp	1
HTH_LUXR + CitB + LuxR_C_like + AAA_16	1
HTH_LUXR + CitB + LuxR_C_like + CsgD + CsgD + sigma70-ECF	1
HTH_LUXR + CitB + LuxR_C_like + CsgD + GerE + sigma70-ECF	1
HTH_LUXR + CitB + LuxR_C_like + FhlA	1
HTH_LUXR + CitB + LuxR_C_like + GAF_2	1
HTH_LUXR + CitB + LuxR_C_like + GerE + GGDEF	1
HTH_LUXR + CitB + Sigma70_r4_2 + sigma70-ECF + CitB	1
HTH_LUXR + CitB + sigma70-ECF	1
HTH_LUXR + CsgD + CitB + GerE + LuxR_C_like	1
HTH_LUXR + CsgD + GerE + LuxR_C_like	1
HTH_LUXR + CsgD + PAS + PAS + PAS	1
HTH_LUXR + CsgD + sigma70-ECF + CheY	1
HTH_LUXR + GerE + CitB + LuxR_C_like + FhlA	1
HTH_LUXR + GerE + LuxR_C_like	1
HTH_LUXR + Glyoxalase + CitB + LuxR_C_like + sigma70-ECF	1
HTH_LUXR + Lrp + HTH_24	1
HTH_LUXR + LuxR_C_like + CitB + AAA_16	1
HTH_LUXR + LuxR_C_like + CitB + Cls	1
HTH_LUXR + LuxR_C_like + CitB + FhlA	1
HTH_LUXR + LuxR_C_like + CitB + GAF + GAF + FhlA	1
HTH_LUXR + LuxR_C_like + CitB + GAF_3	1
HTH_LUXR + LuxR_C_like + CitB + GerE + sigma70-ECF	1
HTH_LUXR + LuxR_C_like + CitB + PAS_9 + PAS + PAS + PAS	1
HTH_LUXR + LuxR_C_like + CitB + sigma70-ECF	1
HTH_LUXR + LuxR_C_like + CsgD + GerE	1
HTH_LUXR + LuxR_C_like + GerE + CitB + GAF_2	1
HTH_LUXR + LuxR_C_like + GerE + CitB + PAS_4 + PAS + PAS + PAS + sigma70-ECF	1
HTH_LUXR + LuxR_C_like + GerE + CsgD + sigma70-ECF	1
HTH_LUXR + LuxR_C_like + HTH_LUXR + CitB	1
HTH_MARR + MarR + MarR + HTH_ARSR	1
LuxR_C_like + CitB + CsgD	1
LuxR_C_like + HTH_LUXR + CitB + CsgD	1
LysR_substrate + PBP2_LTTR_substrate + HTH_LUXR + LuxR_C_like + CitB + LysR	1
MEDS + HTH_LUXR + CitB + LuxR_C_like	1
MhpC + HTH_LUXR + LuxR_C_like + CitB	1
OmpR + REC + Response_reg + REC + HTH_LUXR + CsgD	1
PAS + CitB + HTH_LUXR + PAS + PAS	1
PAS + CsgD + PAS + PAS	1
PAS + PAS + CsgD	1
PAS + PAS_3 + CitB + HTH_LUXR + PAS + PAC	1
PRK08295 + RpoE + Sigma70_r2	1
PRK09639 + RpoE + sigma70-ECF + Sigma70_r4 + Sigma70_r4_2	1
PRK09652 + RpoE + sigma70-ECF + Sigma70_r2 + Sigma70_r4	1

RpoE + sigma70-ECF + Sigma70_r4 + Sigma70_r4	1
RpoE + sigma70-ECF + Sigma70_r4_2 + Sigma70_r4	1
Sigma70_r4 + Sigma70_r4 + sigma70-ECF	1
Sigma70_r4 + sigma70-ECF + Sigma70_r4_2	1
Sigma70_r4_2	1
TolB + HTH_LUXR + CitB + GerE + LuxR_C_like + PD40	1
Whib + CsgD	1
rplJ + Ribosomal_L10 + RplJ + Ribosomal_L10 + Sigma70_r4_2	1
sensory_box + CitB + HTH_LUXR + PAS_9 + PAS + LuxR_C_like + PAS + GerE + PAC	1
sigma70-ECF + HTH_24	1
sigma70-ECF + RpoE + HTH_LUXR + LuxR_C_like	1
sigma70-ECF + RpoE + Sigma70_r2 + PKS_PP	1
sigma70-ECF + RpoE + Sigma70_r4	1
sigma70-ECF + RpoE + Sigma70_r4 + Sigma70_r4	1
sigma70-ECF + RpoE + Sigma70_r4_2	1
sigma70-ECF + Sigma70_r4_2 + sigma70-ECF	1

Supplementary Table S8. Table showing details of GO IDs involved in biological processes and molecular functions.

GO IDs	***Biological Processes***
GO:0006355	regulation of transcription, DNA-templated
GO:0006351	transcription, DNA-templated
GO:0000160	phosphorelay signal transduction system
GO:0006352	DNA-templated transcription, initiation
GO:0006412	translation
GO:0006457	protein folding
GO:0009307	DNA restriction-modification system
GO:0015031	protein transport
GO:0042254	ribosome biogenesis
GO IDs	***Molecular Functions***
GO:0003677	DNA binding
GO:0003700	transcription factor activity, sequence-specific DNA binding
GO:0016987	sigma factor activity
GO:0000155	phosphorelay sensor kinase activity
GO:0016301	kinase activity
GO:0003824	catalytic activity
GO:0008984	protein-glutamate methylesterase activity
GO:0000287	magnesium ion binding
GO:0003723	RNA binding
GO:0003726	double-stranded RNA adenosine deaminase activity
GO:0003735	structural constituent of ribosome
GO:0003899	DNA-directed 5'-3' RNA polymerase activity
GO:0005524	ATP binding
GO:0008233	peptidase activity
GO:0009036	Type II site-specific deoxyribonuclease activity
GO:0016740	transferase activity
GO:0016887	ATPase activity
GO:0046914	transition metal ion binding
GO:0070180	large ribosomal subunit rRNA binding

Supplementary Table S9. Table showing list of all ligands predicted using COACH software available in I-TASSER package among LuxR regulators (70) in Gram-positive bacteria.

Ids	Ligands
A0A0A8EXN6	Manganese (+2); Trifluoroberyllate (-1); c-di-GMP; Nucleic Acid; Xenon; D-Tartaric Acid; Azide
A0A0B2B580	N-(3-Oxo-octanal-1-yl)-homoserine lactone; Nucleic Acid; Homoserine Lactone; Glycerol
A0A0B2B5N7	N-Decanoyl-DL-homoserine lactone; Nucleic Acid; Homoserine lactone; Glycerol
A0A0B2B6K0	N-3-Oxo-dodecanoyl-L-homoserine; N-(3-Oxo-octanal-1-yl)-homoserine lactone; Nucleic Acid; Glycerol
A0A0B2BK36	N-3-Oxo-dodecanoyl-L-homoserine; Nucleic Acid; Homoserine lactone; Glycerol; 3-oxooctanoic Acid
A0A0B2BQK8	N-3-Oxo-dodecanoyl-L-homoserine lactone; Nucleic Acid; Homoserine lactone; Glycerol
A0A0C1YIC3	N-(3-Oxo-octanal-1-yl)-homoserine; Nucleic Acid; N-3-Oxo-dodecanoyl-L-homoserine lactone; Homoserine lactone; Glycerol; 3-Oxooctanoic Acid
A0A0F7VZU8	Manganese (+2); c-di-GMP; Nucleic Acid; Xenon; Glycerol; Platinum (+2)
A0A0H4U956	Magnesium (+2); Trifluoroberyllate (-1); c-di-GMP; Nucleic Acid; D-Tartaric Acid; Xenon; N-Cyclopropyl-N-(cis-4-cyclopropyl-4-hydroxycyclohexyl)-4-[(1S)-2,2,2-trifluoro-1-hydroxy-1-methylethyl]benzamide
A0A0K1F8Q7	Beta-L-Fucose; Magnesium (+2); Copper dimer; Nucleic Acid; Dimethyl sulfoxide; Aspargine; Xylopyranose; Gamma-butyrolactone
A0A0K2YH36	Manganese (+2); Trifluoroberyllate (-1); k-mer; Xenon
A0A0M7QJ27	Magnesium (+2); Nucleic Acid; c-di-GMP; Trifluoroberyllate (-1); Xenon; Azide; D-Tartaric Acid; N-Cyclopropyl-N-(cis-4-cyclopropyl-4-hydroxycyclohexyl)-4-[(1S)-2,2,2-trifluoro-1-hydroxy-1-methylethyl]benzamide
A0A0M8ZBK1	N-(3-Oxo-octanal-1-yl)-homoserine; N-3-Oxo-dodecanoyl-L-homoserine; Nucleic Acid; 3-Oxooctanoic Acid; Glycerol; c-di-GMP; D-Tartaric Acid
A0A0M9YNQ3	Calcium (+2); Nucleic Acid; k-mer; Trifluoroberyllate (-1); c-di-GMP; D-Tartaric Acid; N-Cyclopropyl-N-(cis-4-cyclopropyl-4-hydroxycyclohexyl)-4-[(1S)-2,2,2-trifluoro-1-hydroxy-1-methylethyl]benzamide; Glycerol
A0A0N0B8Y7	N-(3-Oxo-octanal-1-yl)-homoserine; 2,4-Dibromo-6-({[(2-chlorophenyl)carbonyl]amino}methyl)phenyl 2-methylbenzoate; Nucleic Acid; Homoserine lactone; Glycerol; 3-Oxooctanoic Acid
A0A0N0U2C6	N-(3-Oxo-octanal-1-yl)-homoserine; 2,4-Dibromo-6-({[(2-chlorophenyl)carbonyl]amino}methyl)phenyl 2-methylbenzoate; Nucleic Acid; Glycerol; 3-Oxooctanoic Acid; Homoserine lactone
A0A0P4RE30	Magnesium (+2); Trifluoroberyllate (-1); Nucleic Acid; c-di-GMP; k-mer; Azide
A0A0U3KDU7	Calcium (+2); c-di-GMP; Trifluoroberyllate (-1); D-Tartaric Acid; Nucleic Acid; N-Cyclopropyl-N-(cis-4-cyclopropyl-4-hydroxycyclohexyl)-4-[(1S)-2,2,2-trifluoro-1-hydroxy-1-methylethyl]benzamide; L-Proline; Azide
A0A0U5HHD0	Manganese (+2); c-di-GMP; Trifluoroberyllate (-1); Nucleic Acid; Glycerol; Calcium (+2)
A0A135GLC1	(5S)-2-{[(1S)-1-(2-Fluorophenyl)ethyl]amino}-5-methyl-5-(trifluoromethyl)-1,3-thiazol-4(5H)-one; 4-Cyclopentyl-N-[(1S,3R)-5-oxidanyl-2-adamantyl]-2-[[(3S)-oxolan-3-yl]amino]pyrimidine-5-carboxamide; Magnesium (+2); Trifluoroberyllate (-1); k-mer; NADPH; Nucleic Acid; c-di-GMP; D-Tartaric Acid; NADP; (5R)-2-[(2-Fluorophenyl)amino]-5-(1-methylethyl)-1,3-thiazol-4(5H)-one
A0A178WYG5	Trifluoroberyllate (-1); c-di-GMP; Deoxycholic acid; k-mer; 2-{4-[(4-Amino-2-methylpyrimidin-5-yl)methyl]-3-methylthiophen-2-yl}ethyl trihydrogen diphosphate
A0A1B2H2W2	Magnesium (+2); Trifluoroberyllate (-1); Nucleic Acid; k-mer; Xenon; c-di-GMP
A0A1C4JLS0	Magnesium (+2); Trifluoroberyllate (-1); Nucleic Acid; k-mer; 2-Amino-2-hydroxymethyl-propane-1,3-diol; Glycerol; Acetate ion; c-di-GMP;D-Tartaric Acid
A0A1C5CV71	Calcium (+2); c-di-GMP; Trifluoroberyllate (-1); D-Tartaric Acid; Nucleic Acid; Platinum (+2); Glycerol; k-mer; Acetate ion; Magnesium (+2); Glycerol; 2-Amino-2-hydroxymethyl-propane-1,3-diol
A0A1D3DY12	Calcium (+2); c-di-GMP; Nucleic Acid; Trifluoroberyllate (-1); D-Tartaric Acid;

	Azide; 2-Amino-2-hydroxymethyl-propane-1,3-diol; Glycerol; Acetate ion; Magnesium (+2)
A0A1G9ZMY9	Nucleic Acid; c-di-GMP; Trifluoroberyllate (-1); Magnesium (+2); GDP; 1-Palmitoyl-2-linoleoyl-SN-glycero-3-phosphocholine; L-Proline
A0A1K1YMA4	c-di-GMP; Manganese (+2); Trifluoroberyllate (-1); Nucleic Acid; 2-Amino-2-hydroxymethyl-propane-1,3-diol; Glycerol; Acetate ion; Magnesium (+2); Azide
A0A1K2FK71	Magnesium (+2); c-di-GMP; Trifluoroberyllate (-1); Nucleic Acid; D-Tartaric Acid; Azide; 2-Amino-2-hydroxymethyl-propane-1,3-diol; Glycerol; Acetate ion
A0A1M6UHK3	Manganese (+2); Trifluoroberyllate (-1); k-mer; Platinum (+2); Glycerol; c-di-GMP; 3-Chloro-4-({(2R)-4-[4-fluoro-2-(trifluoromethyl)phenyl]-2-methylpiperazin-1-yl}sulfonyl)benzamide; D-tartaric Acid
A0A1M7J3Q0	Trifluoroberyllate (-1); Manganese (+2); c-di-GMP; Nucleic Acid; D-Tartaric Acid; 2-Amino-2-hydroxymethyl-propane-1,3-diol; Glycerol; Acetate ion; Magnesium (+2)
B6VC61	2,4-Dibromo-6-({[(2-chlorophenyl)carbonyl]amino}methyl)phenyl 2-methylbenzoate; Nucleic Acid; Glycerol; Homoserine lactone
B6VC62	2,4-Dibromo-6-({[(2-chlorophenyl)carbonyl]amino}methyl)phenyl 2-methylbenzoate; Nucleic Acid; Glycerol; Homoserine lactone
C3JQB5	Nucleic Acid; Phosphate ion; Calcium (+2); D-Fructose-6-phosphate; Zinc (+2); 3-Cyclohexyl-1-propylsulfonic acid; Propanol
D9VGZ0	Trifluoroberyllate (-1); Nucleic Acid; k-mer; Calcium (+2); c-di-GMP; 2-Amino-2-hydroxymethyl-propane-1,3-diol; Glycerol; Acetate ion; Magnesium (+2); Guanosine-5'-RP-alpha-thio-triphosphate; Glycerol; Calcium (+2);
A6QJN1	Manganese (+2); Trifluoroberyllate (-1); Peptide; Nucleic Acid; Xenon; c-di-GMP; Magnesium (+2)
O07528	Magnesium (+2); Trifluoroberyllate (-1); Peptide; Azide; Manganese (+2); k-mer; c-di-GMP; D-Tartaric Acid; Xenon
A0A010ZIZ4	Magnesium (+2); Trifluoroberyllate (-1); Nucleic Acid; c-di-GMP; 3-Cyclohexyl-1-propylsulfonic Acid; Imido diphosphate; D-Tartaric Acid
A0A098B3B4	Magnesium (+2); Trifluoroberyllate (-1); Peptide; Nucleic Acid; c-di-GMP; Xenon; Guanosine-5'-RP-alpha-thio-triphosphate; Sulfate ion; Chloride (1); Azide; k-mer
A0A076I8Y8	Magnesium (+2); Trifluoroberyllate (-1); Peptide; Xenon; Nucleic Acid; c-di-GMP; 3-Cyclohexyl-1-propylsulfonic acid
A0A096KPL9	Manganese (+2); Trifluoroberyllate (-1); Peptide; Magnesium (+2); Imido diphospahte; c-di-GMP; Nucleic Acid; Xenon
A0A023X7C8	Magnesium (+2); Trifluoroberyllate (-1); Nucleic Acid; c-di-GMP; Xenon; D-Tartaric Acid; Imido diphosphate; Guanosine-5'-RP-alpha-thio-triphosphate; Sulfate ion; Chloride (-1); Magnesium (+2);
A0A1M4SAD4	Manganese (+2); Trifluoroberyllate (-1); Peptide; N-Cyclopropyl-N-(cis-4-cyclopropyl-4-hydroxycyclohexyl)-4-[(1S)-2,2,2-trifluoro-1-hydroxy-1-methylethyl]benzamide; c-di-GMP; Xenon; k-mer; Imido diphosphate; 2-Amino-2-hydroxymethyl-propane-1,3-diol; Glycerol; Acetate ion; Magnesium (+2)
A0A099I597	Magnesium (+2); Trifluoroberyllate (-1); Peptide; Nucleic Acid; c-di-GMP; D-Tartaric Acid; k-mer; Tetrafluoroberyllate (-2); Guanosine-5'-RP-alpha-thio-triphosphate; Sulfate (-1); Chloride (-1); Trifluoroberyllate (-1); Peptide; c-di-GMP; Magnesium (+2); Nucleic Acid;
A0A0K2SG57	Calcium (+2); Trifluoroberyllate (-1); Peptide; c-di-GMP; Magnesium (+2); 3-Cyclohexyl-1-propylsulfonic acid
A0A075KF52	Magnesium (+2); Trifluoroberyllate (-1); Peptide; Nucleic Acid; N-Hexanoyl-L-homoserine lactone; c-di-GMP; D-Tartaric Acid; k-mer; 3-Cyclohexyl-1-propylsulfonic acid
A0A1G4FFF5	Manganese (+2); Trifluoroberyllate (-1); Peptide; Nucleic Acid; Xenon; c-di-GMP; Magnesium (+2); Beryllium difluoride; k-mer; D-Tartaric Acid; Sulfate ion
P54662	Magnesium (+2); Trifluoroberyllate (-1); Peptide; Nucleic Acid; Xenon; c-di-GMP; Sulfate ion; Imido Diphosphate
Q5HLK6	Manganese (+2); Trifluoroberyllate (-1); Peptide; Nucleic Acid; c-di-GMP; Magnesium (+2); 3-Cyclohexyl-1-propylsulfonic acid
A0A011RLP8	Magnesium (+2); Trifluoroberyllate (-1); Peptide; Nucleic Acid; 4-[(1R,2S)-3-(4-Benzylpiperidin-1-yl)-1-hydroxy-2-methylpropyl]phenol; Imido diphosphate; Xenon

A0A095XU09	Manganese (+2); Trifluoroberyllate (-1); Peptide; c-di-GMP; k-mer; D-Tartaric Acid; Alpha-D-glucose; 3-Cyclohexyl-1-propylsulfonic acid; Beryllium difluoride
E0NP90	Manganese (+2); Trifluoroberyllate (-1); Peptide; Xenon; Azide; Beryllium difluoride; 2-Amino-2-hydroxymethyl-propane-1,3-diol; Glycerol; Acetate ion; Magnesium (+2); Imido diphosphate; Phomopsin A; c-di-GMP; D-Tartaric Acid
A0A0K8JB09	Manganese (+2); Trifluoroberyllate (-1); Peptide; Magnesium (+2); Nucleic Acid; k-mer; Imido diphosphate; c-di-GMP; D-Tartaric Acid
E7N200	Manganese (+2); Trifluoroberyllate (-1); Peptide; Imido Diphosphate; Nucleic Acid; c-di-GMP; Magnesium (+2)
B3XSK4	Magnesium (+2); Trifluoroberyllate (-1); Peptide; Xenon; Beryllium difluoride; Nucleic Acid; 3-Cyclohexyl-1-propylsulfonic acid; c-di-GMP; D-Tartaric Acid; Imido diphosphate
A0A016QL51	Manganese (+2); Trifluoroberyllate (-1); Peptide; Nucleic Acid; c-di-GMP; Magnesium (+2); Beryllium diphosphate; Imido diphosphate
D7CTR9	Magnesium (+2); Trifluoroberyllate (-1); Peptide; Nucleic Acid; N-Hexanoyl-L-homoseine lactone; Imido diphosphate; 3-Cyclohexyl-1-propylsulfonic acid
A0A0B0QFU8	Magnesium (+2); Trifluoroberyllate (-1); Deoxycholic Acid; Xenon; Nucleic Acid; 3-Cyclohexyl-1-propylsulfonic acid; c-di-GMP
D6TC16	Magnesium (+2); Trifluoroberyllate (-1); Peptide; 3-Cyclohexyl-1-propylsulfonic acid; c-di-GMP; Guanosine-5'-RP-alpha-thio-triphosphate; Sulfate ion; Chloride (-1); Imido diphosphate; Nucleic Acid; D-Tartaric Acid
D1CB60	Magnesium (+2); Trifluoroberyllate (-1); Peptide; c-di-GMP; Nucleic Acid; 3-Cyclohexyl-1-propylsulfonic acid; k-mer; Guanosine-5'-RP-alpha-thio-triphosphate; Acetate ion; Chloride (-1)
A0A1M3BBK8	Manganese (+2); Trifluoroberyllate (-1); Peptide; Nucleic Acid; Xenon; 3-Cyclohexyl-1-propylsulfonic acid; Magnesium (+2); c-di-GMP; D-Tartaric Acid; k-mer
A0A0K0GBP7	Manganese (+2); Trifluoroberyllate (-1); Peptide; Nucleic Acid; Xenon; Imido Diphosphate; Magnesium (+2)
B2A755	Magnesium (+2); Trifluoroberyllate (-1); Peptide; Nucleic Acid; Xenon; c-di-GMP; Imido Diphosphate; 3-Cyclohexyl-1-propylsulfonic acid; Sulfate ion
A0A174T0B8	Manganese (+2); Trifluoroberyllate (-1); Peptide; Magnesium (+2); N-Hexanoyl-L-homoserine lactone; Nucleic Acid; NADP; k-mer
A0A0J6X083	Magnesium (+2); Trifluoroberyllate (-1); Peptide; c-di-GMP; Imido Diphosphate; Guanosine-5'-RP-alpha-thio-triphosphate; Sulfate ion; Chloride (-1); Manganese (+2); 3-Cyclohexyl-1-propylsulfonic acid; Nucleic Acid
M1YZZ2	Magnesium (+2); Trifluoroberyllate (-1); Peptide; Nucleic Acid; Manganese (+2); k-mer; Glycerol; c-di-GMP; D-Tartaric Acid
A0A1F9ZS08	Magnesium (+2); Trifluoroberyllate (-1); Peptide; Xenon; Imido diphoshate; Beryllium difluoride; Nucleic Acid; k-mer
A0A1F2XDC8	Magnesium (+2); Trifluoroberyllate (-1); Peptide; Nucleic Acid; Xenon; c-di-GMP; Manganese (+2); c-di-GMP
A0A010YIL7	Magnesium (+2); Trifluoroberyllate (-1); Peptide; Imido diphosphate; c-di-GMP; Nucleic Acid; 4-(4-Chlorophenoxy)-N-[(3S)-2-oxotetrahydrofuran-3-yl]butanamide
A0A076JGU5	Magnesium (+2); Trifluoroberyllate (-1); Peptide; k-mer; Nucleic Acid; Xenon; c-di-GMP; Guanosine-5'-RP-alpha-thio-triphosphate; Sulfate ion; Chloride ion
A0A068NFZ6	Manganese (+2); Trifluoroberyllate (-1); Peptide; c-di-GMP; Magnesium (+2); Nucleic Acid; 3-Cyclohexyl-1-propylsulfonic acid

Supplementary Table S10. List of all the conserved residues in 11 LuxI containing Gram-positive bacteria sequences against *V. fischeri* LuxI sequence extracted using MAFFT alignment tool and viewed using Jalview software.

Conserved residues	Position with gap	% consensus	position w.r.t *V. fischeri*
L	84	67	24
R	85	100	25
Y	86	42	26
V	91	50	28
F	92	92	29

W	98	75	35
E	109	83	44
D	111	100	46
Y	113	58	48
D	114	83	49
V	136	58	65
G	138	92	67
R	141	100	70
L	142	67	71
L	151	83	72
P	152	83	73
T	153	75	74
L	159	58	80
P	164	50	85
P	176	75	94
P	179	50	97
I	182	58	99
E	184	83	101
R	187	83	104
V	190	58	107
L	221	75	125
I	225	50	129
A	229	33	133
G	233	83	137
E	302	33	150
R	303	58	151
R	307	58	155
G	317	83	164

Supplementary Table S11. List of all the conserved residues in 800 LuxR containing Gram-positive bacteria sequences against *V. fischeri* LuxR sequence extracted using MAFFT alignment tool and viewed using Jalview software.

**Conserved residues**	**Position with gap**	**% consensus**	**position w.r.t *V. fischeri***
L	2027	79.3	183
T	2084	52.7	184
R	2150	66	186
E	2166	74.4	187
E	2189	41.6	189
L	2267	59.4	191
G	2376	85.3	197
I	2417	75.2	203
L	2433	77.4	207
S	2496	64.8	210
T	2507	82.5	213
V	2528	71.5	214
H	2563	59.7	217
K	2672	72.7	224
L	2678	61.3	225
R	2820	72.5	230
G	3066	37.8	241

Supplementary Table S12. List of the LuxI and LuxR proteins of Gram-positive bacteria used in amino acid composition, motif, domain, gene ontology and clustering analyses.

Proteins	UniProt IDS
LuxI (11)	A0A011RMS3, A0A0M8ZFU8, A0A0N0BAZ2, A0A0N0B7G7, D6TN77, A0A1L4BYL7, A0A0B2BR17, A0A1B1WGF3, A0A1H6EQS4, A0A0N0B975, A0A0B7MAK3
LuxR (800)	A0A0U0N4G9, A0A174E7I3, A0A087D1Z9, A0A059N1S1, A0A087A214, A0A0U0JZZ2, A0A0T0MFQ9, A0A076JND1, A0A0L6JW54, A0A096KM83, A0A136Q3Y9, A0A068NP91, A0A101NH56, A0A089ZL43, A0A151BX90, A0A067LWT9, A0A096KYP7, A0A143X2Y5, A0A0N1KGX5, A0A075EYH4, A0A096LNE6, A0A0L6VYY7, A0A0G4DWD3, A0A0C6ER42, A0A0X8JEF5, A0A100JJX1, A0A143XEN7, A0A021VXE9, A0A023WZK8, A0A033UVV7, A0A163HEV4, A0A101HAJ5, A0A139WQ51, A0A094RSU7, A0A0M8KM93, A0A172ZFQ9, A0A0L0QL86, A0A0W1JLU7, A0A086ZFS8, A0A0M9UC00, A0A126ZWA7, A0A0N0I4N4, A0A143X5E7, A0A140KDT0, A0A087EFN7, A0A198AEH4, A0A086ZT23, A0A023X6Q4, A0A160T6E6, A0A0C5BZ65, A0A087ATB7, A0A163KMV4, A0A0F6FJA8, A0A140KDJ6, A0A0Q8VNJ9, A0A0B5RYL7, A0A034UJZ5, A0A068NJE5, A0A087ECW5, A0A0B2AJG4, A0A0F3RRU6, A0A0T9EN19, A0A0D4BYM4, A0A087D7Y9, A0A0D6YNY4, A0A068NJC8, A0A0D0PQA4, A0A098BG17, A0A172T7A8, A0A0A8VVR6, A0A143QDX5, A0A172ZC60, A0A0F5AIK5, A0A0T1W7K0, A0A0P6X9T5, A0A0J5R124, A0A0Q5I327, A0A072N7Q5, A0A0D6KZ17, A0A087D6Q5, A0A0B5ETQ2, A0A0R2BEV1, A0A0D4DRU1, A0A0N7YM16, A0A0J8I4U4, A0A0N0TYD1, A0A0K3BMY2, A0A098B3B4, A0A061N5Y2, A0A0F0H2Y8, A0A0N1NEZ2, A0A174CP94, A0A154V4P3, A0A163YXF0, A0A1A0SDJ1, A0A0B2B285, A0A126Y1N6, A0A161S1M7, A0A0P7ZM07, A0A101S2R5, A0A0T8KT33, A0A0C1U2R5, A0A0A7KHT9, A0A109RGL7, A0A0L6VZB8, A0A163Z3V0, A0A178WZZ4, A0A162FXS5, A0A0J5GTL6, A0A0B2BK36, A0A0C1U1T1, A0A0U3KDU7, A0A178WWI5, A0A1A0KBT2, A0A0U9HD19, A0A0D0PHX6, A0A078MBI5, A0A143XAT5, A0A0K2CU67, A0A173YWW1, A0A010YWQ0, A0A089KZB1, A0A0L0WEE5, A0A174JUQ2, A0A0M3WNA3, A0A011AW57, A0A0B2B1S5, A0A0M4GG94, A0A090ZWV3, A0A060JH75, A0A010ZY94, A0A0K8JHB1, A0A0P9CAN1, A0A173Z7N2, A0A117QM02, A0A169PPY9, A0A124E6M1, A0A0B5R7S3, A0A094PE74, A0A136NYG5, A0A0B6EYY3, A0A0C1RML4, A0A0B5ID94, A0A0A1MJ53, A0A174KB65, A0A010PN22, A0A132PB53, A0A0B5EX96, A0A089LYM9, A0A075JUE7, A0A170PEL4, A0A109MWW8, A0A179IRC8, A0A117RZX9, A0A136NVX6, A0A0F0L8H4, A0A0B7MK55, A0A0H1SUX8, A0A0U3KQE0, A0A0H2KKM6, A0A0Q5JH85, A0A0X3UQZ4, A0A0U1KS47, A0A0M1NV77, A0A0P6XZK0, A0A109IHJ6, A0A0K9Z0B6, A0A0Q6VG75, A0A132MNW5, A0A173XTP5, A0A0Q6LE79, A0A117LKD7, A0A0F5HJA8, A0A0F5VMY0, A0A0K2REY0, A0A068NSS5, A0A0S6UF37, A0A0P9CID6, A0A0F7NFY7, A0A075JU19, A0A0L8QTS9, A0A0R1MNN6, A0A136NYQ9, A0A0U4WYH1, A0A0Q6UX39, A0A0S2PFA0, A0A0Q6HER8, A0A0Q5VNN8, A0A0P6XVH6, A0A034UAA5, A0A0M8THE4, A0A1A3GN77, A0A100JRC4, A0A0H1ABG7, A0A0Q9SA32, A0A166VRI3, A0A0Q8YQW8, A0A0T5ZJT3, A0A101RJS7, A0A0Q9NFD7, A0A117MPG5, A0A0C1BWA6, A0A0N1HEA8, A0A0Q9JCY4, A0A0G8AU43, A0A0N0HWZ6, A0A0D8BIK6, A0A061NJ29, A0A176QFG3, A0A0C9SJH4, A0A0R1QHX5, A0A0K3BTQ4, A0A023X4P0, A0A062WPZ2, A0A0Q9JLF4, A0A0T1T284, A0A0B2BLK4, A0A0S2YUT3, A0A0M9UBA5, A0A0P9FAJ5, A0A0V7ZUX1, A0A0B2BLZ5, A0A140E7S4, A0A0S7BHT3, A0A0N0MKT5, A0A0C1YIC3, A0A0P7BVU3, A0A0Q8Y236, A0A172X234, A0A0M8ZML2, A0A0B2BQX3, A0A081NXM2, A0A0M0KNE3, A0A0L6VYQ1, A0A135GLC1, A0A0U9H2D0, A0A0B5F2I8, A0A0N9HZB1, A0A177PXE2, A0A0F0GLJ4, A0A0L0JT16, A0A0J5R3W6, A0A0F2PQF4, A0A0S4QWM2, A0A0Q4RMN2, A0A0P9DEM2, A0A0B2ARD2, A0A0U0Z5K9, A0A0M8T8Y6, A0A0P6YSS1, A0A0C1U4E8, A0A0R1U1A4, A0A0A0BB95, A0A0Q9PZY2, A0A086N1G7, A0A0A0JUS8, A0A0Q9MLU9, A0A1A2P2H2, A0A0B2BK03, A0A0B2YL89, A0A0B2BHJ1, A0A0B2B4I9, A0A094PG95, A0A0B2BB17, A0A0B2B5N7, A0A0B2B6K0, A0A0B2BES1, A0A0B2B605, A0A0N0SX27, A0A0L6CFL9, A0A160KRK0, A0A0X3VGM3, A0A0Q9R450, A0A0X3UMA5, A0A0K3B3F9, A0A061A8I7, A0A0A3J6Y4, A0A1A0MLV2, A0A0X3SRU7, A0A0Q9K1Z7, A0A0W7W6C3, A0A0M0A7Q4, A0A0B8NP95, A0A0D0X0M2, A0A087AZR2,

	A0A109UGI9, A0A0G7ZN25, A0A0H5SL01, A0A0Q9N5G6, A0A0Q4M1V6, A0A162E4M1, A0A0G3HL81, A0A139CNY5, A0A0F4KKS9, A0A0L0JWQ6, A0A069CT21, A0A0A3HYV4, A0A0A1CUB5, A0A117KJI5, A0A0R2BJG8, A0A0Q7BME3, A0A0M4QMN4, A0A033UHJ3, A0A0S8BEA3, A0A0K1F3B1, A0A073BA84, A0A0B5F2J1, A0A0D6AJW2, A0A0F0H481, A0A0A8FP17, A0A0A0NN59, A0A0F0GJB4, A0A117KXX7, A0A151D7K8, A0A0Q9K0K6, A0A0C2QM10, A0A0N0B7W1, A0A0K1JNK4, A0A0N0B875, A0A0B6SWY9, A0A0U3PGJ5, A0A0U3QEP5, A0A140KZV2, A0A0Q8Q0V3, A0A166P488, A0A0A0BDM0, A0A081EEP4, A0A0A0J4C0, A0A0K8Q416, A0A101SM71, A0A0E1L200, A0A0E2HIJ6, A0A0Q6G5I9, A0A168JI87, A0A193JU72, A0A0Q4CMX2, A0A023X1F8, A0A117QSK3, A0A022L5Y0, A0A0B5AYJ3, A0A0Q6R9Y0, A0A0D8HEF7, A0A151BWF9, A0A0N0B8Y7, A0A087AJB3, A0A124H1H5, A0A0F5N5M6, A0A0M0F061, A0A0L0BGL9, B6VC61, A9WJU6, B7GGX6, C4FFF6, A4PHL5, B5IMZ0, E4LJK7, C6D7K8, A7NI42, B9L143, D8K3S6, A9AVS1, A9B8N6, A0A1F8QL47, A0A1I9Z791, A0A1C6PEH0, F7UZ49, G5CJ99, C7GAX2, A0A1C0ZWA7, A5ZQJ6, E4NIF4, B0REE9, A0A1D7QTM9, E1VZP8, A5UY27, F3B4P6, A0A1C6L916, C2V2T9, F6FQJ3, G8TS53, F7UW40, F7UZW4, F7UW28, D6Y058, A0A1F8QS82, E6LJ92, A0A1F2UG98, F7UU47, A0A1F2UNA8, C6PMI5, G9XRB3, A0A1F8L576, D9WZ37, A5UPE8, F7UYB1, A4F7V0, G8TZF1, E6JZW6, A0A1J4SF32, A9WTC2, A0A1E8CUN8, D2B3R4, D0WEE2, A0A1F8LXH0, A0A1H6JBI6, A0A1F6QE52, A0A1F8MTW9, A0A1H6EVC6, A0A1C4ZSP9, A0A1F8LQI6, A0A1F2WZL7, A0A1C4NUT7, D9UZT3, G9PKA3, A0A1C6AXD8, A0A1D2KG87, A0A1J1LCV6, G2NWX1, B4WPS3, D0YSN2, D3R622, A0A1F6QDB5, A0A1H1QCB5, A0A1C4VEZ3, D6AXM2, D0YQC0, C8W5L4, G2G221, A0A1C6S915, A0A1E4IBL2, A0A1B3XKW2, A0A1B5ZFT2, A0A1F1VUV2, A0A1F8L809, D6Y0G1, A0A1F9ZS08, G1WK02, A0A1F8Q323, D5PA66, A0A1C3P5W0, A0A1K3D7A0, C7Q4D3, A0A1D7QTU8, A0A1C5K0L0, D9V9T1, D2K2B7, A0A1F6Q1M0, A0A1C6UVN9, A0A1H0JUC0, H1CYP9, A0A1C5AF54, C6WE86, H5TKQ9, A0A1J5A0K1, A0A1C5TPB1, D2AUY9, A0A1F8MT95, A0A1C6DUP1, A0A1H4GUQ0, H6CGG2, A0A1C6I6Y7, D6TT67, E6TRA7, G8WPC7, A0A1C4RF08, A0A1H5VSH7, D7CTM8, F5SIW0, B7KLS0, D0LDI8, D5UCN6, B2JB82, B5GV68, F1TAV9, D9WM95, D2PNP9, C4RND9, D6TGX0, F3NFX7, C5BWG9, C9ZBF6, D3PVC4, C0ZSB6, E9UPH4, A0A1K1RRR6, E4RLE4, C6WIR5, H0RG82, A0A1B2HMC0, C4RMU4, F4FB17, A0A1H6EU71, A0A1K0FQG4, A0A1D2VY43, A0A1G9RR55, E1QXZ5, A0A1H1Z239, A0A1H1RC56, A0A1H2MHU0, A0A1C6SI55, A0A1F1AZ75, D9XCR4, C4RFX4, A0A1H6ER37, F4CKY1, A0A1H2IKI6, H1ZZ97, F8FKL5, A0A1F6QDA1, A0A1H1YEY0, A0A1C6SB53, A0A1E9AEW6, A0A1H5VAY8, A0A1C4QS66, A0A1F2XXD9, A0A1G4VEP0, A0A1A9DM71, A0A1B1BI48, A0A1F8MUA8, A0A1F8PGV9, A0A1C5UTU0, A0A1A9BAQ0, A0A1K1Y803, A0A1E4HWU4, E6S9C7, G4HNQ2, A0A1C5CGZ6, A0A1E4I578, A0A1C4MPP4, A0A1C5FBT4, G4FJI2, H6RJW2, C7N2J8, C9Z8X8, F0PS05, A0A1K1T6B8, D9VW08, A0A1J0US29, A0A1C6M987, C7N7C7, A0A1C5KTF3, A0A1C5ES70, A0A1G8PB28, A0A1G8RIS5, F5LI40, D2SNE2, A0A1H1U3V8, A8L5P7, A0A1C4RUT2, B7DVE9, A0A1C3NZG5, A0A1H2N000, D3F5A4, A0A1C4NXV2, A0A1A9DNI2, F8IGP0, A0A1G4VUY5, B2GCH9, E0D209, A0A1F2WCU2, A0A1C5E718, A0A1H1U5X5, A0A1F8P8T6, F6ENW3, A0A1J0AE40, A0A1J6Y2G6, A0A1C4WU09, A0A1H1X1I8, A5UUE9, A0A1E5K213, B5H0M7, A0A1B9CP10, D4L1E6, A0A1E2SM40, H0E7X8, A0A1C6H5K2, A0A1E2W6H1, A0A1A3N1N9, D3G0Z2, A0A1G7C0R5, A0A1F1Z1L3, D9VVL8, A4FHF0, A0A1J0G226, C1ARD3, C1A2X3, H0E6L2, H0K4I2, A0A1M8XTR7, H5X1M1, A0A1M9JU27, A0A1A9BIA4, F8F995, A0A1M7KLM5, A0A1E8VWF3, A0A1C6QL47, A0A1M5XNL1, A0A1M9JTZ8, A0A1M9LTL8, A0A1M7HBA5, A0A1L8QDF2, A0A1M5BED1, B9L4H6, A0A1L9DRM6, D6Y0A0, A0A1F6RI30, A0A1C4AMG1, A0A1M6QFN0, A0A1L7NQB7, A0A1L7EMZ6, A0A1M5TYE3, A0A1B7VGB4, A0A1M3BA61, A0A1M3BJG2, A0A1M6SSJ9, A0A1M4E827, A0A1N3XHS4, A0A1M4S4U5, A0A1M7DGJ6, A0A1C5DXR9, A0A1A9BT10, A0A1C5GK81, A0A1C5EGH9, A0A1H0T4I5, A0A1C6RLN0, A0A1M5RCA4, A0A1M6YLU5, A0A1M3AS96, E0I6D1, A0A1N6NB14, A0A1M5F9R7, A0A1L4BX59, A0A1K0FA93, D9TMV3, A7NKI6, A0A1L2ZP06, D9Y0U4, D6A912, F3ZFG3, H0R3N6, A0A1M6F4K9, A0A1M7TX32, A0A1M5YWY9, F2R685, A0A1D8TQT5, A0A1M5BD83, A0A1M5TR22, C2JZP1, P72781, Q93KX3, Q0ICL8, Q8FN06, Q0RML2,

	Q67Q89, Q67Q81, S3XYK2, Q2JK46, Q2JLS7, M3FIL7, S0EV32, U9VYF2, Q55104, K9VEW5, U5QK54, Q8EMF0, M2Q822, Q0RHM5, S4MGA0, T4BX91, K0YUP0, R6DUZ9, T2ITS7, W7RKY0, U5QKE5, W5IIQ8, W1TK86, R7HAL3, W9DNL6, W5W721, W5IGL3, K4QYT4, R7DTK7, R7ABU2, L0EI42, U2QUY1, I4EQF8, R4L795, S3BAX6, K4R6X7, I4ESI5, J4K788, M7AJS2, W9GD20, R9L9S1, W2F0R3, H8E4T6, U5YR10, W9B6K4, I4EIH2, Q1MX86, V6IZ62, I9A9R6, N6X2V1, I7G450, R5VNH4, R7YAX8, U6SMW9, S0EWC0, R7LPK1, I3DY29, W7ST35, V6JHT9, U4QWP3, R6ST01, R7MR06, W7SPC0, R2SQW0, M0QK75, K9VBA6, S9TZD0, R6DMF0, L7KML8, S6CDW9, I4FY38, M1MBZ7, M3TSS9, M1ZK39, I5AT62, S6G053, I0WNT6, K9P845, V6JZC3, R4SJG3, K9SLT3, L7L031, I8APT4, M7AIU3, R4T6K9, J0NKR3, W7CNI1, W4VAJ2, W4Q7E7, Q2B8K6, K6VTN2, M5E269, W7S8F0, R1CFT1, I0LBX3, M1YWH2, J8P7L0, M3VF05, I0GPL2, K0K819, W5W9P5, R7D914, I4EV40, M2YK20, K0J809, T4V9U5, R7D7P6, W7W4S0, I4EX01, U1X323, U2T2X2, W7S7X1, R4T1C8, K0K4G1, R1G5G8, K0K2C7, K0K3U3, R1IG72, I1D0S3, J3B3D6, R1IBE1, V6KA12, U9VW55, U9VNI5, K4RAP9, V6JTR0, I0GVF7, W6K005, S3BJT7, K9ELL3, W5WDK5, K1EAE5, W5WDL2, K8GTT2, V6JNL1, L1MMA6, W7IVS3, J1RAL1, J5UUJ2, L7F3J7, R5L593, W9FUJ2, L0J6E9, I0R019, K6X8N4, I9A0J9, M3C1N5

Supplementary Table S13. List of the LuxI and LuxR containing proteins of Gram-positive bacteria, and Gram-negative bacteria used in phylogenetic analyses.

Group	Regulator	Protein IDs
Gram-positive bacteria	LuxI	A0A011RMS3, A0A0B2BR17, A0A0B7MAK3, A0A0M8ZFU8, A0A0N0B7G7, A0A0N0B975, A0A0N0BAZ2, A0A1B1WGF3, A0A1H6EQS4, A0A1L4BYL7, D6TN77
BLAST hits	LuxI	WP_071896677.1, WP_039344015.1, WP_084721451.1, KTS10845.1, WP_015927656.1, WP_056522302.1, KTS11796.1, WP_064440131.1, WP_018716450.1, WP_053066202.1, WP_077750786.1
Gram-positive bacteria	LuxR	D7CTR9, A0A068NFZ6, A0A178WYG5, A0A010YIL7, A0A075KF52, A0A0K8JB09, A0A1K1YMA4, A0A1K2FK71, A0A016QL51, B3XSK4, A0A1B2H2W2, A0A0U5HHD0, A0A0A8EXN6, A0A1M7J3Q0, A0A0U3KDU7, D6TC16, M1YZZ2, B6VC61, B6VC62, A0A0M9YNQ3, A0A1D3DY12, A0A0M7QJ27, P54662, A0A0K0GBP7, A0A0B0QFU8, A0A1F9ZS08, E0NP90, A0A0N0U2C6, A0A0M8ZBK1, A0A0N0B8Y7, A0A135GLC1, A0A1C4JLS0, A0A011RLP8, A0A099I597, A0A174T0B8, A6QJN1, Q5HLK6, A0A1C5CV71, A0A1M4SAD4, A0A0J6X083, A0A0K2SG57, A0A010ZIZ4, A0A1M3BBK8, D1CB60, A0A096KPL9, A0A095XU09, A0A1G4FFF5, A0A023X7C8, A0A076I8Y8, E7N200, A0A098B3B4, D9VGZ0, A0A0B2B5N7, A0A0H4U956, A0A0B2B6K0, A0A0B2B580, A0A0B2BQK8, A0A0B2BK36, A0A1M6UHK3, A0A0F7VZU8, C3JQB5, A0A1G9ZMY9, A0A0P4RE30, A0A1F2XDC8, B2A755, A0A0K2YH36, A0A076JGU5, A0A0C1YIC3, A0A0K1F8Q7, O07528
BLAST hits	LuxR	WP_049724721.1, AHU89214.1, WP_009207225.1, WP_063728485.1, WP_037615761.1, WP_002610814.1, WP_077510809.1, OJY48092.1, WP_044335533.1, WP_053238709.1, WP_031335348.1, WP_077445499.1, SEC91280.1, WP_055945165.1, EAZ59603.1, WP_003119559.1, WP_018925932.1, WP_078320909.1, WP_047184658.1, WP_067985891.1, WP_067060808.1, WP_029146894.1, WP_029361113.1, KIU27256.1, AIQ94021.1, WP_056241970.1, WP_077381447.1, WP_072694598.1, WP_044619495.1, OGT25317.1, WP_056148495.1, OEU49755.1, WP_015860242.1, SCY57304.1, WP_015904281.1, OGP48145.1, OLC71345.1, OPL07345.1, WP_035854665.1, KPJ68470.1, WP_040394504.1, ANU66337.1, WP_059739663.1, WP_069252139.1, AEA63556.1, KKL43669.1, WP_046548602.1, WP_071332319.1, ODN64030.1, WP_029080541.1, SFO30912.1, SFH67863.1, WP_027581039.1, WP_028354053.1, OGA19779.1, SDZ00979.1, WP_012096589.1, WP_049187717.1, WP_053997513.1, ESK45491.1, WP_044156617.1
